# Development of a Novel Polyleucine‐Based Immunopotentiator for Subunit Vaccines Against Group A Streptococcus

**DOI:** 10.1002/smll.202511459

**Published:** 2026-02-17

**Authors:** Lantian Lu, Jazmina L. Gonzalez Cruz, Rabina Giri, Yuexiao Zhang, Sahra Bashiri, Leslie C. Domínguez Cadena, Shuhang Li, Zeinab G. Khalil, Waleed M. Hussein, Jingwen Wang, Jolynn Kiong, Xuehan Lu, Jiahui Zhang, Miao Lu, Wenbin Huang, Wei Yang Kong, Ummey Jannatun Nahar, Prashamsa Koirala, Zhong Guo, Patricia Walden, Antje Blumenthal, Jakob Begun, Kirill Alexandrov, Istvan Toth, Rachel J. Stephenson, Mariusz Skwarczynski

**Affiliations:** ^1^ School of Chemistry and Molecular Biosciences The University of Queensland Brisbane Queensland Australia; ^2^ Department of Microbiology and Immunology Indiana University School of Medicine Indianapolis Indiana USA; ^3^ Frazer Institute Translational Research Institute The University of Queensland Brisbane Queensland Australia; ^4^ Mater Research Institute Translational Research Institute The University of Queensland Brisbane Queensland Australia; ^5^ Australian Institute for Bioengineering and Nanotechnology The University of Queensland Brisbane Queensland 4072 Australia; ^6^ Institute for Molecular Bioscience The University of Queensland Brisbane Queensland Australia; ^7^ School of Pharmacy The University of Queensland Brisbane Queensland Australia; ^8^ School of Biology and Environmental Science Queensland University of Technology Brisbane Queensland Australia

**Keywords:** adjuvant discovery, group A streptococcus, polyleucine, subunit vaccines, vaccine immunopotentiators

## Abstract

Mixture‐based vaccine formulations offer distinct advantages by eliminating the need for chemical conjugation or complex encapsulations, thereby streamlining preparation, enhancing versatility, and reducing cost and production time. In this study, we report the design of a novel polypeptide‐based self‐assembling adjuvant where a water insoluble 15‐mer polyleucine (L_15_) is coupled with a polylysine unit (K_6_). As a nano adjuvant, L_15_K_6_ markedly enhances antigen uptake by dendritic cells, promotes antigen‐presenting cell maturation (CD40, CD80/86, and MHC‐II), and upregulates Toll‐like receptor expression, particularly Toll‐like receptor 4 in dendritic cells. Bulk RNA sequencing further reveals robust NF‐κB pathway activation by L_15_K_6_. When co‐delivered with peptide or protein antigens derived from Group A Streptococcus, L_15_K_6_ induces robust antigen‐specific IgG response with highly potent antibacterial activity against two clinical Group A Streptococcus strains in vitro. Beyond antigens derived from Group A Streptococcus, L_15_K_6_ also enhances immune responses against model protein antigens such as ovalbumin and bovine serum albumin, highlighting its broad applicability. Notably, a single immunization with L_15_K_6_ outperforms alum adjuvant in eliciting high levels of bovine serum albumin‐specific IgG. These findings establish L_15_K_6_ as a simple yet powerful immunopotentiator with significant promise for the development of broadly applicable subunit vaccines.

## Graphical abstract

Self‐assembling amphiphilic peptide L_15_K_6_ is used as a novel immunoadjuvant for the development of a Group A Streptococcus vaccine. Robust antigen‐specific IgG responses are generated and manage to kill isolated strains of Group A Streptococcus. Cellular and bulk RNA sequencing data reveals that L_15_K_6_ regulates immune responses potentially through TLR and NF‐κB signaling pathways.

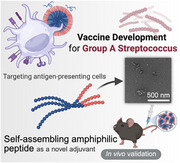

## 1 Introduction

Subunit vaccines are gaining importance in immunization research as they offer many advantages, like precise compositions, targeted immune responses, favorable safety profiles, and simple and rapid production methods. However, subunit antigens, such as peptides and proteins, are less immunogenic than whole pathogens and require immunopotentiators to enhance efficacy [1]. Although clinical immunopotentiators are limited and restricted by regulatory agencies, such as the FDA, that license adjuvants as vaccine components rather than separate compounds or formulations. Adjuvants approved for vaccine formulations include: a) aluminum salts [2]; b) emulsion‐based adjuvant systems AS01, AS03, AS04 and MF59 [3]; c) Toll‐like receptor (TLR)‐based immunopotentiators, such as TLR4 agonists, monophosphoryl lipid A (MPL) [4]; TLR9 agonist CpG 1018 [5]; and d) saponin‐based adjuvant Matrix‐M [6]. Other adjuvants, such as TLR7 agonist imiquimod, have also been approved for adjuvant therapy but not for vaccines [7]. Despite extensive research, immunopotentiators may not induce the required immune response or are incompatible with different antigens; consequently, development of effective adjuvants with diverse modes of action is critical for improving vaccine efficacy.

Poly(hydrophobic amino acids) (pHAAs) have emerged as potent self‐adjuvanting delivery systems for peptide‐based vaccines [8, 9, 10, 11], and among the different pHAA derivatives, polyleucine has displayed the most promising adjuvanting properties [8, 9]. Polyleucine conjugates, consisting of polyleucine and a peptide antigen, self‐assemble in aqueous solutions into distinct nanoparticles and chain‐like aggregates [8, 9] that induce robust humoral or cellular immune responses without the presence of external adjuvants in different disease models, including Group A Streptococcus (GAS) [8, 9], hookworm [10], human papilloma virus [11], porcine circovirus type 2 [12], SARs‐CoV‐2 [13], tuberculosis [14], and cocaine addiction [15]. Studies have shown that polyleucine conjugates are highly effective for stimulating antigen‐specific immune responses [8, 9, 11, 13, 15], although chemical modification of antigens during conjugation may alter antigen properties and significantly hinder system versatility. Potential association of polyleucine with an antigen through noncovalent interactions prompted the current study to explore the adjuvanticity of soluble polyleucine derivatives as independent adjuvants.

GAS is a Gram‐positive bacterium with a wide spectrum of clinical manifestations ranging from asymptomatic and mild infections, like impetigo, pharyngitis, and scarlet fever, to severe invasive diseases, like pneumonia, streptococcal toxic shock syndrome, and necrotizing fasciitis [16]. GAS infections can also cause autoimmune complications. In 2005, Carapetis et al. estimated that 1.78 million cases of severe GAS infections occur every year globally [17], with GAS‐induced rheumatic heart disease killing over 600 000 people annually [18]. Surges in invasive GAS infections and increased disease severity observed worldwide since mid‐2022, indicate that challenges faced by healthcare systems and public health authorities have adversely affected management and control of GAS infections [19]. However, despite the global health and economic burdens of GAS infections, no commercial vaccines are available to prevent or mitigate GAS infections. Development of prophylactic vaccines against GAS infection is still in the early stage with no candidates reaching phase III clinical trials, even though GAS has been researched for decades [20].

Although multiple GAS antigenic proteins exist, the M protein remains the primary target for vaccine development. However, clinical applications using M protein as a vaccine antigen were abandoned decades ago due to homology between M protein fragments and human cardiac proteins [21, 22]. Still, the M protein is highly immunogenic so many studies have explored and identified antigenic determinants of the M protein that do not induce autoimmunity to serve as vaccine antigens. Recent clinical trials exploring GAS vaccine candidates are largely focused on vaccines containing peptides derived from the M protein [16], such as J8 (QAEDKVKQSREAKKQVEKALKQLEDKVQ), which has gained popularity as a J8‐based conjugate protein in recent Phase I clinical studies [16, 20].

In the current study, PADRE‐J8, consisting of a T cell epitope, pan human leukocyte antigen DR isotype (HLA‐DR) epitope (PADRE; KFVAAWTLKAAA), and B cell epitope J8, was constructed as a chimeric peptide antigen to investigate the immune stimulating effects of novel polyleucine derivatives in vitro and in vivo. These polyleucine derivatives were composed of 15‐mer leucine residues and solubilizing moieties of hydrophilic amino acids with different charges. Solubilizing moieties enhanced solubility of polyleucine in aqueous solutions, conferring amphiphilicity and facilitating self‐assembly into particles.

## 2 Results

### 2.1 Synthesis and Characterization

**FIGURE 1:**
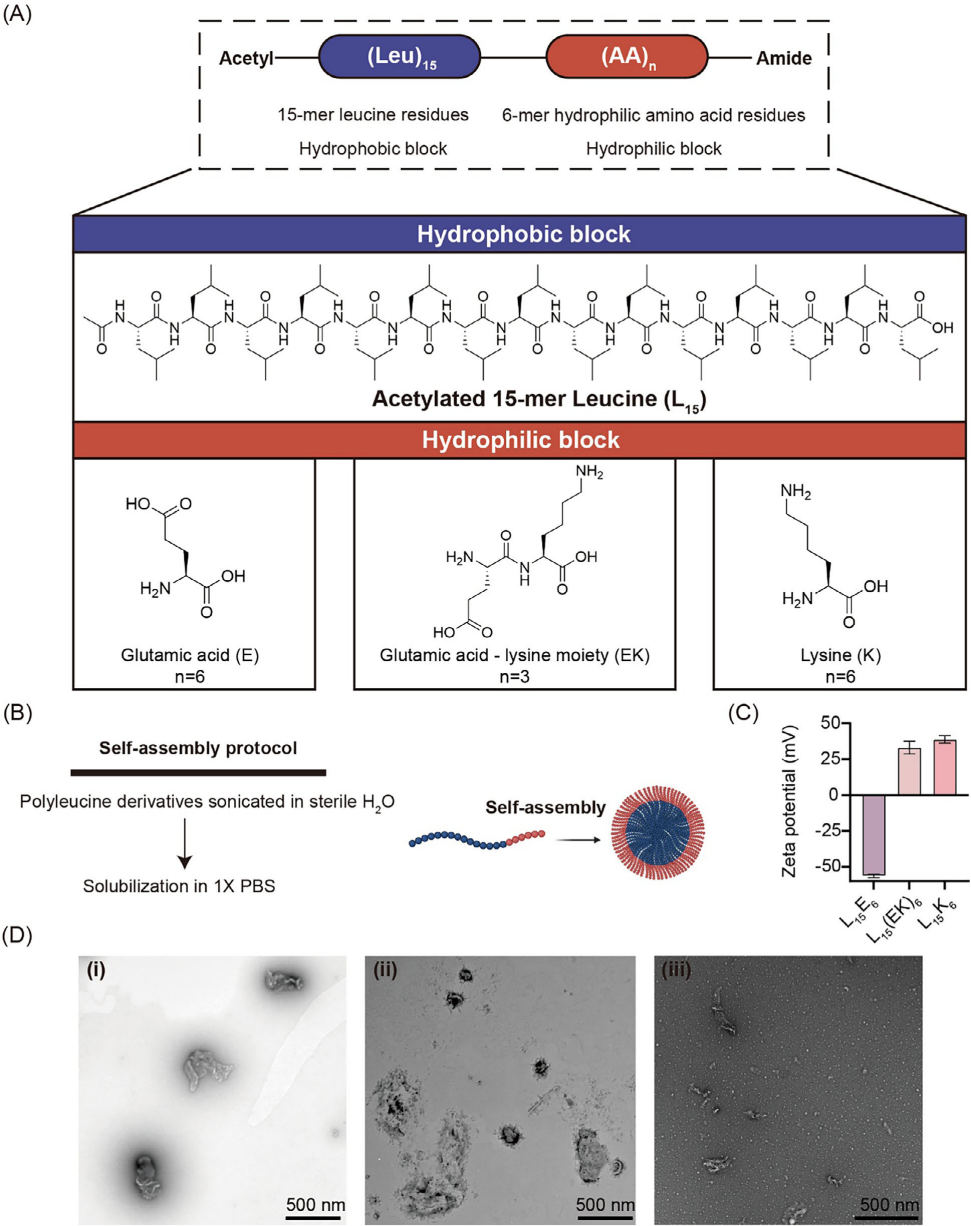
Design of soluble polyleucine derivatives. (A) Amphiphilic polyleucine derivatives composed of fifteen leucine (L_15_) residues and six glutamic acid residues (E_6_), three repeats of glutamic acid‐lysine block (EKEKEK; (EK)_3_), or six lysine residues (K_6_); (B) Polyleucine derivatives self‐assemble in aqueous solutions into particles; (C) Zeta potential of particles formed by different polyleucine derivatives in water. Data shown as mean ± SD; (D) Transmission electron microscopy of particles formed by various polyleucine derivatives: (i) L_15_E_6_; (ii) L_15_(EK)_3_; and (iii) L_15_K_6_.

Polyleucine derivatives were designed to carry solubilizing moieties with negative, neutral, or positive charges at a neutral pH (Figure 1A). Accordingly, > 98% pure L_15_E_6_, L_15_(EK)_3_, and L_15_K_6_, with calculated net charges of −6.0, 0 and +6.0 at pH 7, respectively (Figure S1), were produced using tert‐butyloxycarbonyl (Boc)‐based solid phase peptide synthesis [9] (Figure S2). Self‐assembly was allowed in aqueous solution to form nanoparticles (Figure 1B). Nanoparticles of L_15_E_6_, L_15_(EK)_3_, and L_15_K_6_ displayed different surface charges, with L_15_E_6_ nanoparticles showing a negative surface charge and L_15_(EK)_3_ and L_15_K_6_ showing positive surface charges (Figure 1C).

**FIGURE 2:**
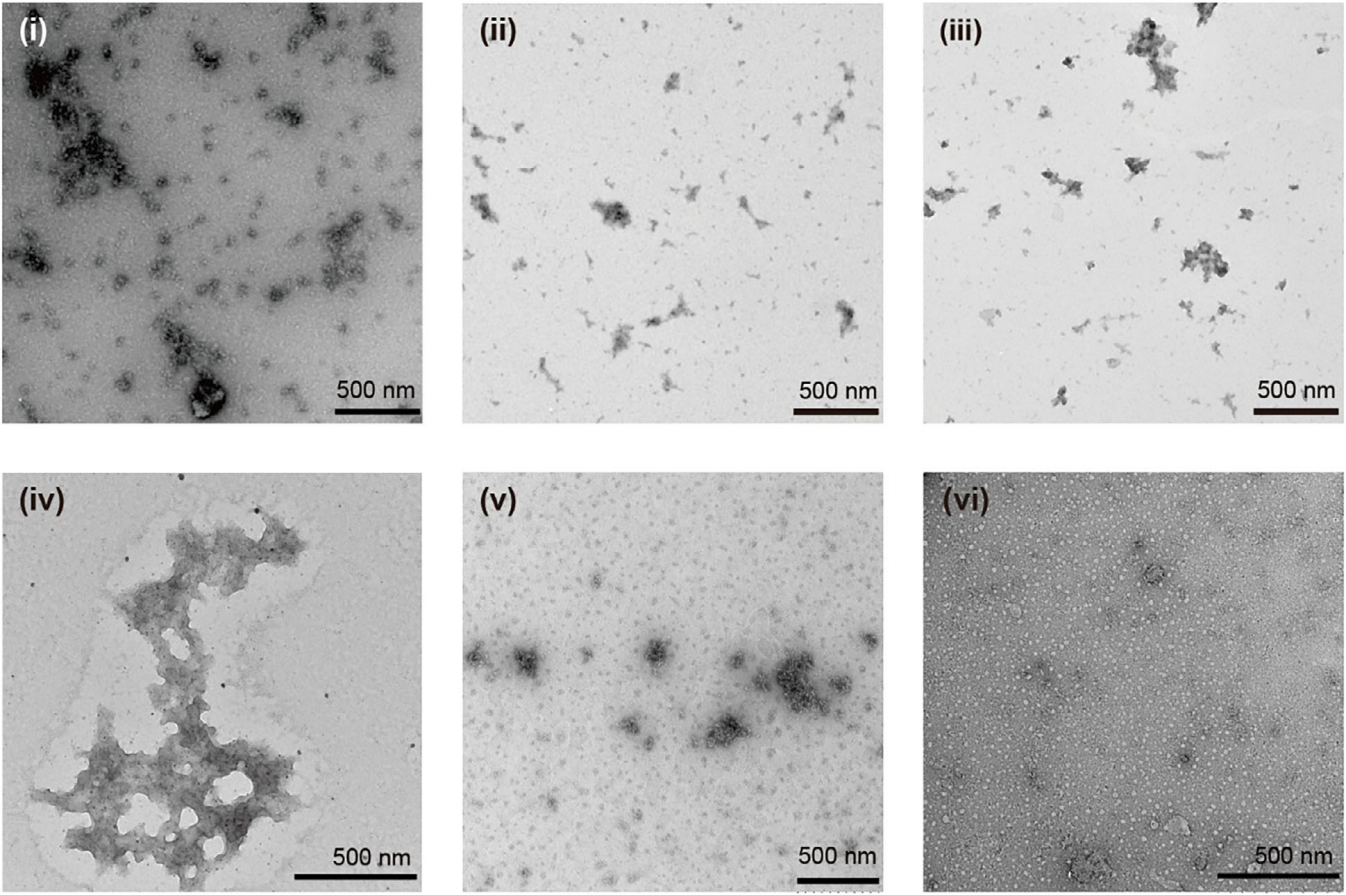
Transmission electron microscopy images of polyleucine derivatives mixed with different antigens. (i) L_15_E_6_+PADRE‐J8; (ii) L_15_(EK)_3_+PADRE‐J8; (iii) L_15_K_6_+PADRE‐J8; (iv) L_15_K_6_+poly(PADRE‐J8); (v) L_15_K_6_+OVA; (vi) L_15_K_6_+BSA.

All particles in Milli‐Q water were assessed through transmission electron microscopy (TEM) (Figure 1D). Formed polyleucine particles were then mixed with solubilized peptide or protein antigens (Figure 2). The size, surface charge, and morphology of polyleucine particles and physical mixtures were analyzed by dynamic light scattering (DLS) and TEM. Particles formed by each polyleucine derivative (L_15_K_6_, L_15_(EK)_3_ and L_15_E_6_) were highly heterogeneous in size. Individual antigens (i.e., PADRE‐J8, poly(PADRE‐J8), OVA, and BSA) all have a tendency to aggregate. Hence, when polyleucine derivatives were mixed with antigens in this study, particles formed with highly polydisperse sizes (Figure 2 i‐vi; Figure S3 and Table S1).

In water, L_15_K_6_ and L_15_(EK)_3_ particles were positively charged, while L_15_E_6_ particles were negatively charged (Figure 1C). The positive charge of L_15_(EK)_3_ particles was most likely due to the presence of trifluoroacetic acid (TFA) salt that favored ionization of amines in lysine residues while inhibiting ionization of glutamic acid side chains. Particles formed with peptide antigen PADRE‐J8 exhibited a positive charge in aqueous solution (30.3 ± 1.8 mV) (Table S1), and when these particles were mixed with L_15_K_6_ particles, a significantly higher positive charge (51.2 ± 1.2 mV) was observed when compared to PADRE‐J8 or L_15_K_6_ alone (30.3 ± 1.8 mV and 38.9 ± 2.6 mV, respectively) (Table S1).

Artificial protein poly(PADRE‐J8), consisting of five copies of PADRE‐J8, was recombinantly expressed by *E. coli* BL21(DE3) (Figure S4) and exhibited a positive charge like PADRE‐J8 (Table S1). In Milli‐Q water, poly(PADRE‐J8) self‐assembled into particles that were more homogenous in size (PDI = 0.24 ± 0.03) than PADRE‐J8 particles (PDI = 0.68 ± 0.10). When poly(PADRE‐J8) was mixed with L_15_K_6_ (Table S1), TEM revealed the formation of large aggregates with a diameter > 500 nm (Figure 2, iv); however, surface charge of poly(PADRE‐J8) (11.7 ± 2.2 mV) was lower than PADRE‐J8 (30.3 ± 1.8 mV), indicating that positively charged amino acids may have embedded inside the protein particles. The resulting particles of L_15_K_6_ and poly(PADRE‐J8) displayed a positive charge (18.0 ± 3.6 mV), reflective of the average of each component (38.9 ± 2.6 mV and 11.7 ± 2.2 mV, respectively) (Table S1). When the negatively charged protein, OVA, was mixed with the positively charged polyleucine derivative, L_15_K_6_, in a 20:1 ratio (m/m), a negatively charged (−18.3 ± 0.9 mV) mixture was produced; however, when the ratio was changed to 4:1, a positive charge was observed (25.4 ± 0.3 mV). A 1:1 mixture of negatively charged BSA (−23.7 ± 0.3 mV) and L_15_K_6_ (38.9 ± 2.6 mV) displayed a positive charge (31.7 ± 1.9 mV), suggesting the presence of L_15_K_6_ on the surface of formed complexes (Table S1).

The secondary structures of polyleucine derivatives and mixtures with antigens were evaluated by Circular Dichroism (CD) spectroscopy (Figure S5). The secondary structure of L_15_K_6_ exhibited a larger proportion of α‐helixes (44%) than L_15_(EK)_3_ (20%) and L_15_E_6_ (20%) (Figure S5). Subsequently, mixing L_15_K_6_ with PADRE‐J8 (L_15_K_6_+PADRE‐J8) produced a higher proportion of alpha helices (36%) in the secondary structure than L_15_(EK)_3_+PADRE‐J8 (25%) or L_15_E_6_+PADRE‐J8 (21%).

### 2.2 Polyleucine Derivatives Facilitated Antigen Uptake by Dendritic Cells

To determine whether polyleucine derivatives of varying charges (L_15_E_6_, L_15_(EK)_3_ and L_15_K_6_) can enhance antigen uptake by antigen‐presenting cells (APCs), such as dendritic cells (DCs), fluorescence‐labelled PADRE‐J8 (5(6)‐carboxyfluorescein‐PADRE‐J8 [23] or cyanine5.5‐PADRE‐J8 [24]; both shorted as fPADRE‐J8) were co‐delivered with L_15_E_6_, L_15_(EK)_3_ or L_15_K_6_ to murine DC cell line DC2.4, with intracellular fluorescence analyzed by confocal microscopy and flow cytometry.

**FIGURE 3:**
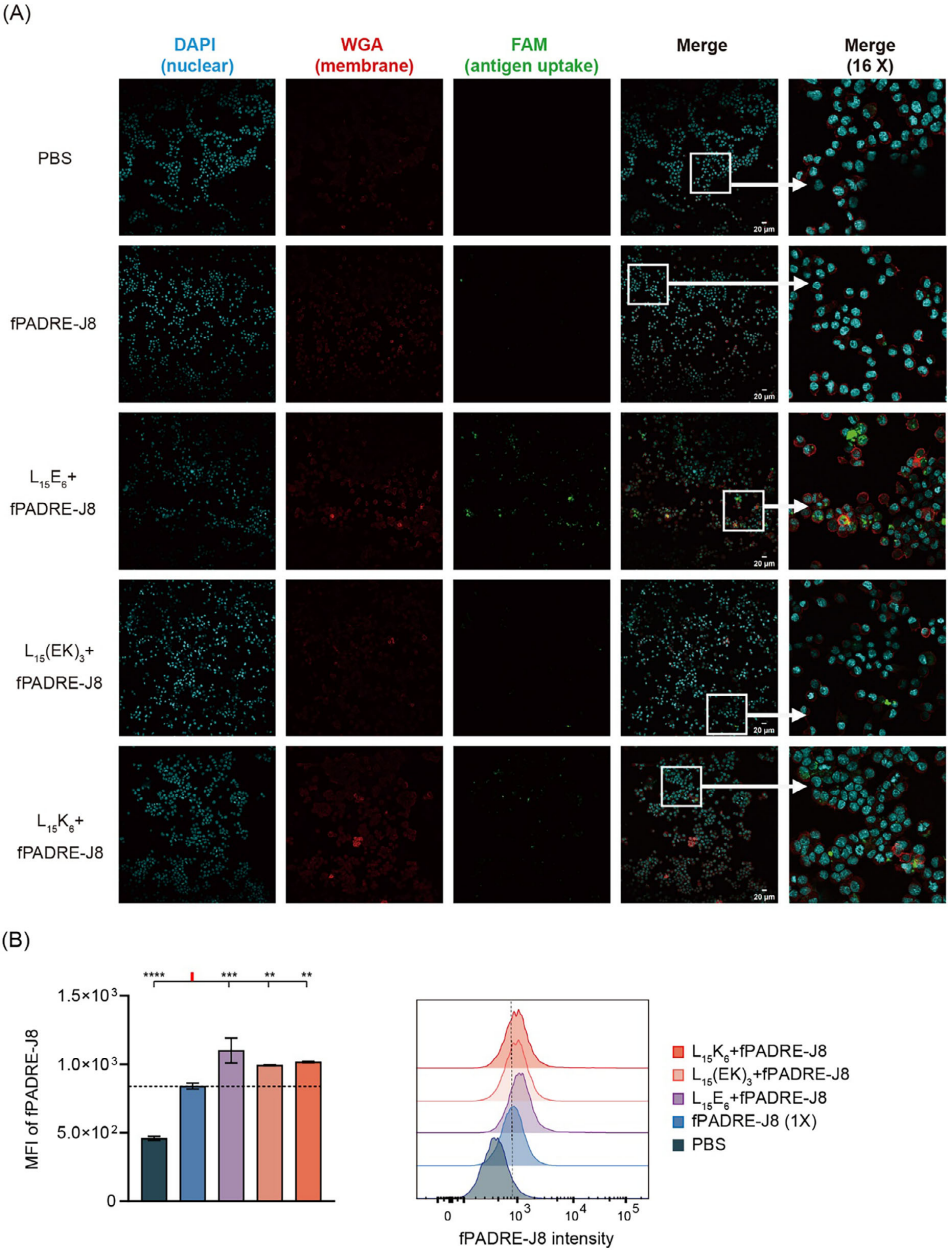
Antigen uptake by dendritic cell line, DC2.4. (A) Confocal microscopy images showing antigen (fPADRE‐J8) uptake by DC2.4 cells; (B) Flow cytometric analysis of cells treated with different vaccine formulations. Data shown as mean ± SD. ns: not significant, *p* > 0.05; *: 0.01 < *p* ≤ 0.05; **:0.001 < *p* ≤ 0.01; ***: 0.0001 < *p* ≤ 0.001; ****: *p* ≤ 0.001. fPADRE‐J8: fluorescence‐labelled PADRE‐J8. Fluorescence used: 5(6)‐Carboxyfluorescein (FAM) or Cyanine 5.5 (Cy5.5). Biological duplicates and triplicates were performed for confocal microscopy and flow cytometry, respectively. MFI: median fluorescence intensity.

Higher fluorescence intensity was observed when DC2.4 cells were co‐cultured with polyleucine co‐delivered fPADRE‐J8 (L_15_E_6_+fPADRE‐J8, L_15_(EK)_3_+fPADRE‐J8, or L_15_K_6_+fPADRE‐J8) than cells co‐cultured with fPADRE‐J8 alone (Figure 3). Confocal microscopy revealed that the highest fluorescence intensity was in cells treated with L_15_E_6_+fPADRE‐J8 (Figure 3A). Similarly, flow cytometry showed that cells treated with polyleucine derivatives+fPADRE‐J8 displayed higher fluorescence intensity than cells treated with fPADRE‐J8 alone, with L_15_E_6_+fPADRE‐J8‐treated cells showing the highest fluorescence intensity (Figure 3B).

### 2.3 Polyleucine Derivatives Enhanced Dendritic Cell Maturation

**FIGURE 4:**
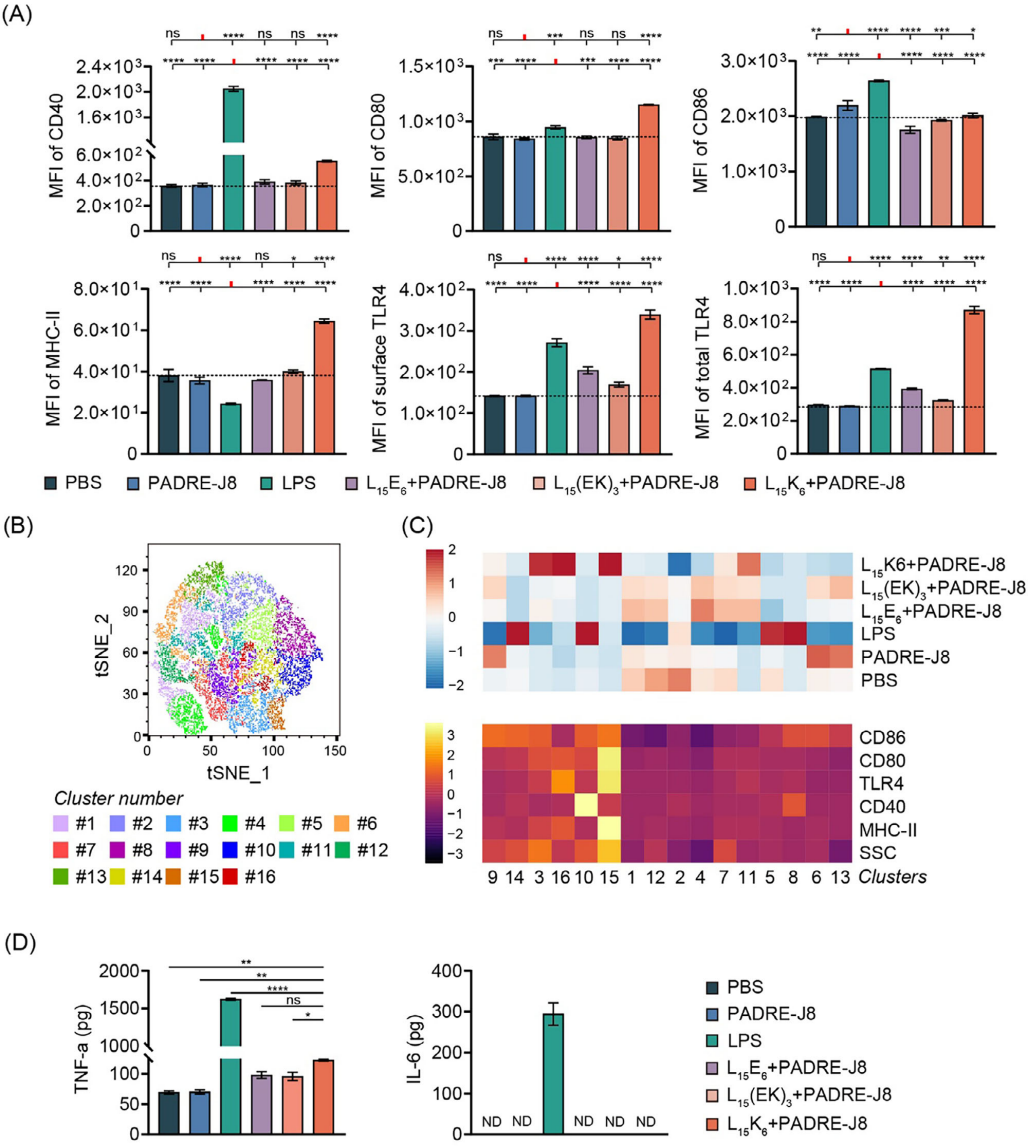
Dendritic cell maturation and cytokine release in response to physical mixture of polyleucine derivatives and PADRE‐J8. (A) Expression of surface CD40, CD80, CD86, MHC‐II, TLR4 and total TLR4 (membrane and cytoplasm) in DC2.4 cells following different treatments for 24 h. Data shown as mean ± SD; (B) UMAP of concatenated cells co‐cultured with different treatments were clustered based on surface maker expression. Sixteen clusters in total were identified; (C) Heatmaps of cluster composition and cluster marker expression. The top heatmap shows the distribution of cells receiving different treatments in each cluster, and the bottom heatmap shows the expression level of each marker in each cluster; (D) Cytokine release by DC2.4 cells following vaccine treatment. Biological triplicates and duplicates were performed for flow cytometry and cytokine ELISA, respectively. Data shown as mean ± SD. SSC: side scatter; ND: not detectable. ns: not significant, *p* > 0.05; *: 0.01 < *p* ≤ 0.05; **:0.001 < *p* ≤ 0.01; ***: 0.0001 < *p* ≤ 0.001; ****: *p* ≤ 0.001.

Dendritic cell maturation markers, CD40, MHC‐II, CD80, and CD86, were evaluated by flow cytometry to determine the maturation status of DC2.4 cells post incubation with antigen or antigen co‐delivered with polyleucine derivatives. PADRE‐J8 alone or co‐delivered with L_15_(EK)_3_ or L_15_E_6_ did not upregulate any maturation markers (Figure 4A); however, PADRE‐J8 co‐delivered with L_15_K_6_ significantly upregulated CD40, CD80, and MHC‐II expression, while the positive control, lipopolysaccharide (LPS), upregulated CD40, CD80, and CD86 expression (Figure 4A). Interestingly, all polyleucine derivatives upregulated expression of Toll‐like receptor 4 (TLR4) like LPS—not only on the plasma membrane but also within the cytoplasm (Figure 4A). These findings indicate that TLR4 molecules were synthesized and translocated to the cell surface following exposure to polyleucine derivatives, with L_15_K_6_ inducing the highest expression of TLR4 expression compared to all other polyleucine derivatives.

Cells treated with vaccine formulations were clustered based on physical side scatter (SSC), to measure granularity and intracellular complexity, and MHC‐II, CD40, CD80, CD86, and TLR4 surface protein expression (Figure 4B). Cells treated with L_15_K_6_+PADRE‐J8 were mostly enriched in cluster 15 and 16, characterized by higher MHC‐II, TLR4, and CD80 expression compared to other clusters (Figure 4B), while cells treated with L_15_E_6_+PADRE‐J8 and L_15_(EK)_3_+PADRE‐J8 were mostly found across clusters 1, 4, 7, 11, 12, characterized by low expression of MHC‐II, CD40, CD80 and CD86. Cells treated with LPS were grouped in clusters 5, 8, 10, 14, with cluster 10 showing the highest CD40 expression.

**FIGURE 5:**
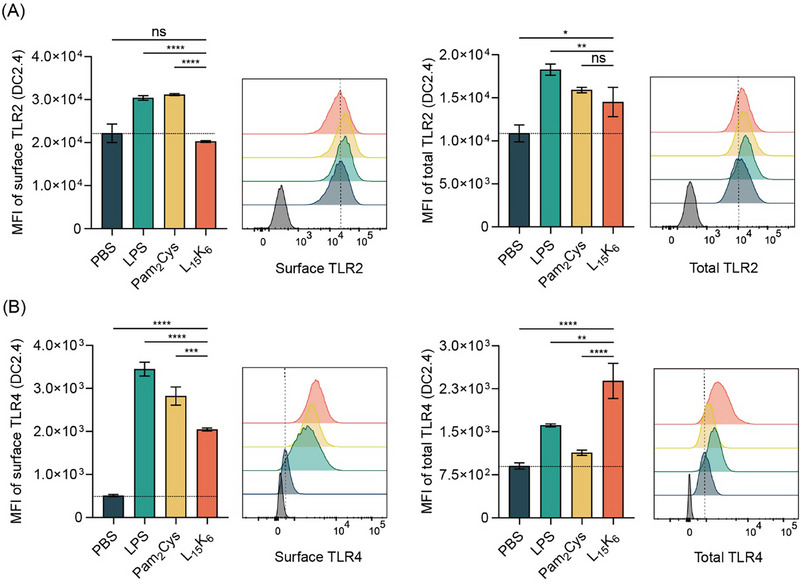
L_15_K_6_ modulated the expression of Toll‐like receptors in DC2.4 cells at a reduced dose. (A) Expression of surface TLR2 or total TLR2 was modulated by L_15_K_6_; (B) Expression of surface TLR4 or total TLR4 was modulated by L_15_K_6_. Gray peak: fluorescence minus one (FMO) sample as control. Data shown as mean ± SD. ns: not significant, *p* > 0.05; *: 0.01 < *p* ≤ 0.05; **:0.001 < *p* ≤ 0.01; ***: 0.0001 < *p* ≤ 0.001; ****: *p* ≤ 0.001.

Cytokine profiling of DC2.4 cell culture supernatants was performed by ELISA (Figures S6 and S8). High secretion of TNF‐α and IL‐6 was induced by LPS. Except for LPS, only L_15_K_6_+PADRE‐J8 induced significantly higher levels of TNF‐α compared to PBS and PADRE‐J8 (Figure 5H). These findings show that LPS strongly activates NF‐κB and JAK/STAT3 signaling pathways, while L_15_K_6_ may only weakly activate NF‐κB pathways. No detectable secretion of IL‐10, IL‐4, or IL‐12 p70 was observed in response to any treatment group (Figure S7).

### 2.4 L_15_K_6_ Modulates TLR and NF‐κB Signaling in Dendritic Cells

L_15_K_6_ at a reduced dose not only modulated the production of TLR4 in DC2.4, but also influenced TLR2 expression (Figure 5A,B). TLR4 agonist LPS and TLR2 agonist Pam_2_CSK_4_ upregulated TLR2 and TLR4 expression both extracellularly and intracellularly (Figure 5A,B). Interestingly, L_15_K_6_ did not affect surface TLR2 expression but significantly enhanced total TLR2 expression, indicating more TLR2 synthesis in the cytoplasm following L_15_K_6_ treatment (Figure 5A). Consistent with earlier finding that L_15_K_6_ (10 µM) upregulated TLR4 expression both extracellularly and intracellularly, a reduced dose (2.5 µM) was also effective (Figure 5B). Interestingly, the central transcription factor of TLR signaling, NF‐κB, was also found largely upregulated by L_15_K_6_ in DC2.4 cells (Figure S8).

### 2.5 L_15_K_6_ Modulated Macrophage Polarization and Cytokine Release

**FIGURE 6:**
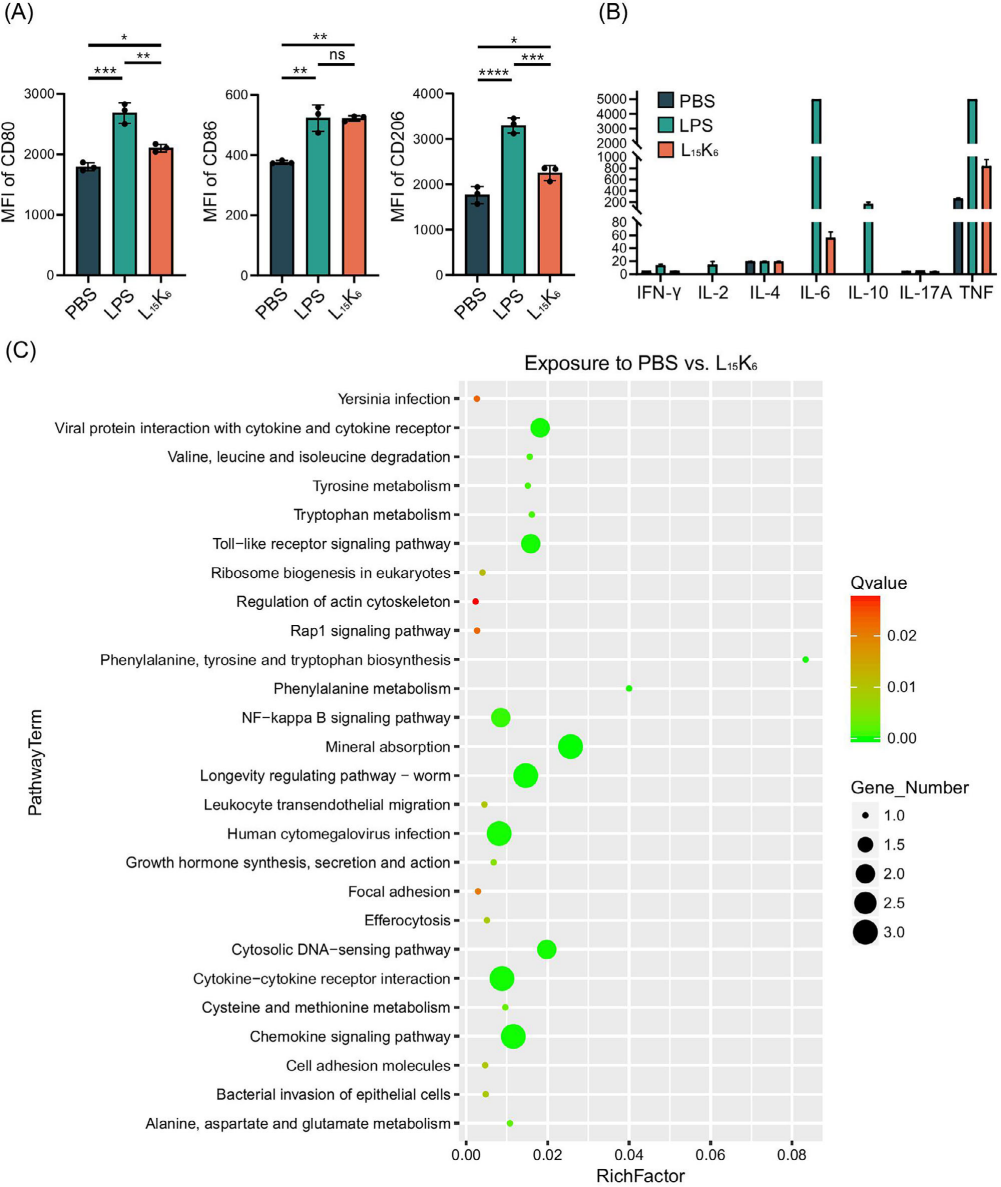
L_15_K_6_ modulated the polarization of macrophages and affect cytokine release, as well as the gene expression in THP‐1 cells. (A) L_15_K_6_ upregulated the expression of CD80, CD86 and CD206, in a similar manner to LPS; (B) L_15_K_6_ stimulated the production of IL‐6 and TNF by macrophages. Data shown as mean ± SD; (C) L_15_K_6_ regulated innate immune signaling and metabolic reprogramming in THP‐1 cells. Significance was assessed based on Q values (Benjamini‐Hochberg adjusted p values controlling for the false discovery rate). Rich factor represents the ratio of the number of differentially expressed genes mapped to a given pathway to the total number of genes annotated in that pathway, reflecting the degree of enrichment.

In addition to modulate the maturation of APCs such as DCs, L_15_K_6_ also showed the ability to regulate the polarization of macrophages (Figure 6A). Unlike LPS that substantially upregulated both M1 and M2 markers, CD80/86 and CD206, L_15_K_6_ only slightly upregulated CD80 and CD206 expression in macrophages while largely increased CD86 expression. Although dramatically less potent in promoting the production of IL‐6 and TNF by macrophages, LPS evidently induced more IL‐6 and TNF secretion by macrophages compared to PBS (Figure 6B).

### 2.6 L_15_K_6_ Regulated Innate Immune Signaling and Metabolic Reprogramming in THP‐1 Cells

To investigate the molecular effects of L_15_K_6_ on innate immune cells, bulk RNA sequencing was performed on L_15_K_6_‐treated THP‐1, human monocytic leukemia cells, compared to PBS‐treated controls (Figure 6C). Up to 12 genes were significantly regulated in THP‐1 cells after exposure to L_15_K_6_ (Figure S9). KEGG pathway enrichment analysis revealed that L_15_K_6_ stimulation led to a robust upregulation of pathways associated with innate immune recognition and inflammatory signaling (Figure 6C). Prominent among those were the Toll‐like receptor signaling pathway, NF‐κB signaling pathway, and the cytosolic DNA‐sensing pathway. This correlated well with the finding in DC2.4 where NF‐κB p65 was upregulated after exposure to L_15_K_6_ (Figure S8). This cross‐cellular concordance indicated a consistent activation of central pathogen‐recognition and cytokine‐production mechanisms [25]. Furthermore, enrichment of cytokine‐cytokine receptor interaction, chemokine signaling, and leukocyte transendothelial migration pathways suggested the induction of chemotactic and immune cell recruitment involvements, overall highlighting that L_15_K_6_ potently engages innate immune sensing machinery and drives a coordinated proinflammatory program across distinct immune cell types.

In addition to immune signaling, exposure to L_15_K_6_ also affected cellular adhesion and cytoskeletal remodeling, including Rap1 signaling, regulation of actin cytoskeleton, focal adhesion, and cell adhesion molecules, consistent with enhanced immune synapse formation and migratory potential [26]. Pathways commonly associated with viral or bacterial infections (e.g., human cytomegalovirus infection, and Yersinia infection) were also enriched, suggesting the shared use of innate signaling modules in host defense rather than real pathogen involvement.

Importantly, L_15_K_6_ also triggered broad enrichment of amino acid metabolic pathways as evidenced by the enrichment amino acid metabolic pathways (valine, leucine, and isoleucine degradation; tyrosine and tryptophan metabolism; phenylalanine, tyrosine and tryptophan biosynthesis; phenylalanine metabolism; cysteine and methionine metabolism; alanine, aspartate and glutamate metabolism). This metabolic reprogramming likely supported the energetic and biosynthetic demands of activated immune cells [27]. Together, these findings demonstrated that L_15_K_6_ has the potential to activate THP‐1 cells, simultaneously engaging canonical innate immune pathways and rewiring cellular metabolism to support immune activation.

### 2.7 L_15_K_6_ Enhanced the Immune Response Mounted by a GAS‐derived Peptide Antigen

**FIGURE 7:**
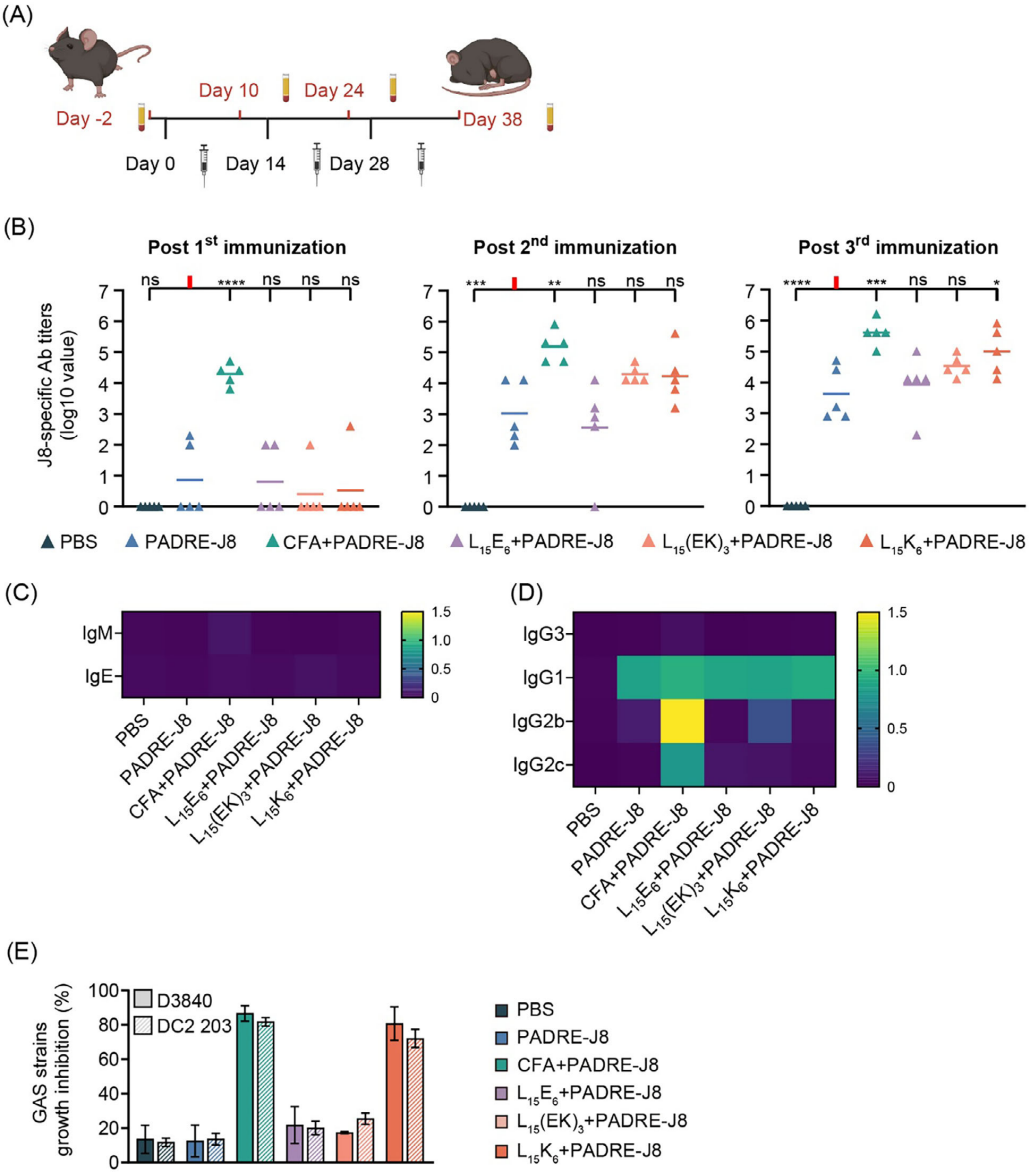
L_15_K_6_ enhanced murine immune responses against GAS‐derived peptide antigen. (A) Vaccination schedule; (B) J8‐specific IgG titers in immunized mice; (C) Level of J8‐speciffic IgM and IgE (shown as mean absorbance, OD450 – OD540, n = 5); (D) Level of J8‐specific IgG subclass (IgG3, IgG1, IgG2b and IgG2c) profiling using pooled sera from each treatment group (performed in triplicates, shown as mean absorbance, OD450 – OD540, n = 3); (E) Bactericidal assay evaluating antibacterial potency of pooled sera from each group of immunized mice against clinically isolated GAS bacteria D3840 and GC2 203 (performed in duplicate, shown as percentage of bacterial growth inhibition). Data shown as mean ± SD. ns: not significant, *p* > 0.05; *: 0.01 < *p* ≤ 0.05; **:0.001 < *p* ≤ 0.01; ***: 0.0001 < *p* ≤ 0.001; ****: *p* ≤ 0.001.

Polyleucine‐based vaccine formulations were explored in C57BL/6J female mice. Briefly, mice were immunized three times subcutaneously with intervals of 14 days (Figure 7A). After three immunizations, higher J8‐specific total IgG titers were detected in mice immunized with L_15_K_6_+PADRE‐J8, L_15_(EK)_3_+PADRE‐J8, and L_15_E_6_+PADRE‐J8 compared to mice immunized with only PADRE‐J8, although no statistically significant differences were observed in J8‐specific IgG titers between mice immunized with PADRE‐J8 and mice immunized with L_15_(EK)_3_+PADRE‐J8 or L_15_E_6_+PADRE‐J8 (Figure 7B).

No antigen‐specific IgM was detected in any group, indicating a short half‐life of IgM (Figure 7C). In addition, antigen‐specific IgE was not detected in any group, indicating that Type I hypersensitivity (IgE‐associated allergic reactions) was unlikely, not even for mice immunized with CFA emulsified PADRE‐J8 (Figure 7C). Pooled sera from each group of mice were profiled for IgG subclasses IgG3, IgG1, IgG2b, and IgG2c (Figure 7D). All sera showed high and comparable levels of antigen‐specific IgG1, except for sera from mice immunized with PBS. Only sera from mice immunized with CFA+PADRE‐J8 showed high levels of antigen‐specific IgG2b and IgG2c, while IgG3 was not detected in any sera samples.

Pooled sera were then analyzed in bactericidal assays where clinical isolates of GAS strains were co‐cultured with the sera. Sera from mice immunized with CFA+PADRE‐J8 or L_15_K_6_+PADRE‐J8 displayed extremely high antibacterial activity that inhibited growth of D3840 and GC2 203 in vitro (Figure 7E). However, sera from L_15_E_6_+PADRE‐J8, L_15_(EK)_3_+PADRE‐J8, and PADRE‐J8 alone exhibited limited or no antibacterial activity against D3840 or GC2 203 in vitro.

### 2.8 L_15_K_6_+PADRE‐J8 Outperformed its Conjugated Equivalent, L_15_‐PADRE‐J8, in Mounting J8‐Specific Immune Responses Against GAS

**FIGURE 8:**
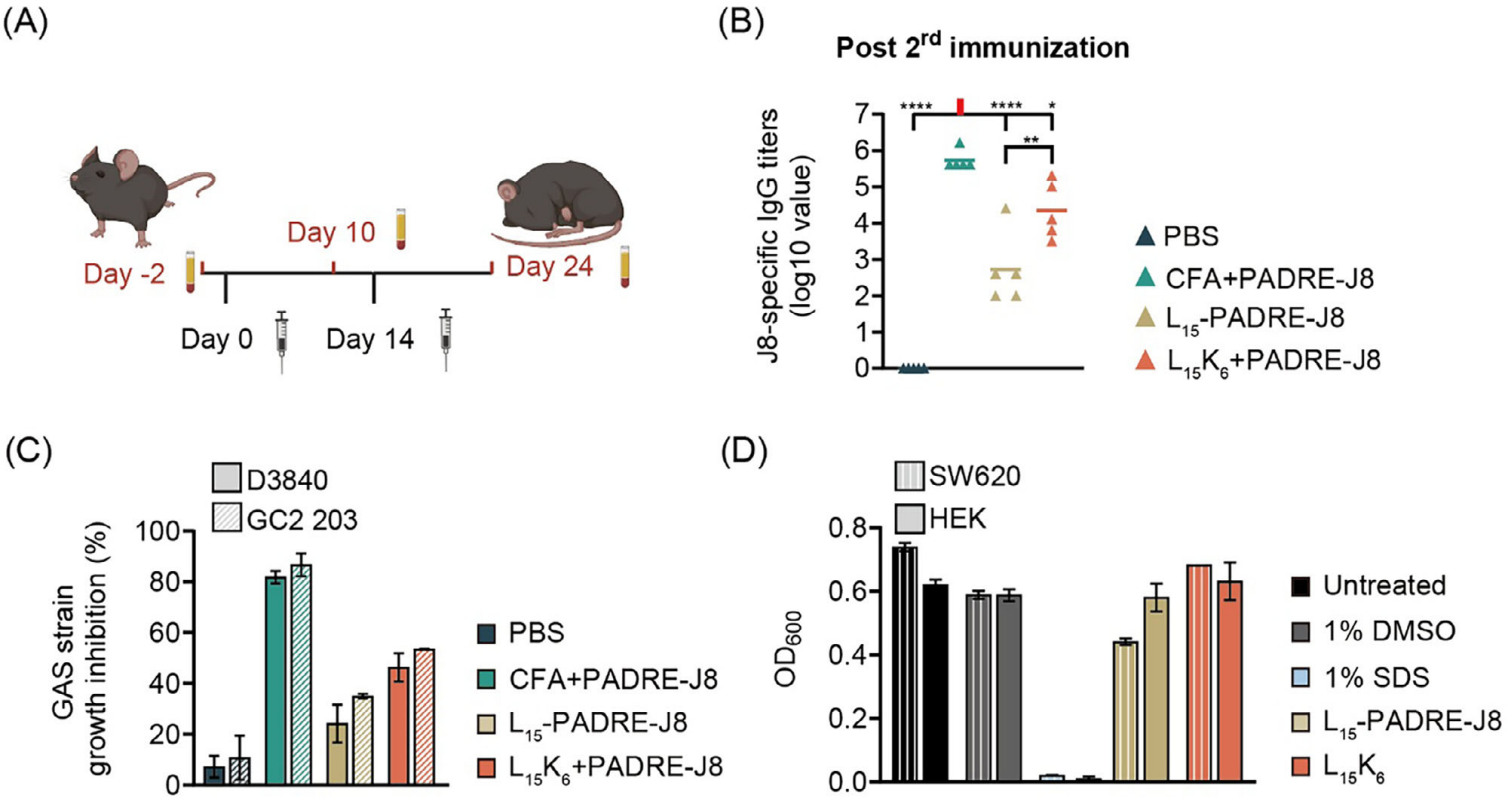
L_15_K_6_+PADRE‐J8 outperformed L_15_‐PADRE‐J8 in efficacy and safety in vivo and in vitro. (A) Vaccination schedule administered to C57BL/6J female mice; (B) J8‐specific total IgG titers induced by different treatment groups after the final (second) immunization; (C) Bactericidal assay evaluating antibacterial potency of pooled sera from each treatment group against clinically isolated GAS bacteria D3840 and GC2 203 (performed in duplicate, shown as percentage of bacterial growth inhibition); (D) Impact of L_15_K_6_+PADRE‐J8 and L_15_‐PADRE‐J8 on SW620 and HEK cell viability by 3‐[4,5‐dimethylthiazol‐2‐yl]‐2,5 diphenyl tetrazolium bromide assay (performed in duplicates); Data shown as mean ± SD. ns: not significant, *p* > 0.05; *: 0.01 < *p* ≤ 0.05; **:0.001 < *p* ≤ 0.01; ***: 0.0001 < *p* ≤ 0.001; ****: *p* ≤ 0.001.

L_15_K_6_+PADRE‐J8 was compared with L_15_‐PADRE‐J8 in vivo to determine if conjugation was required to generate robust J8‐specific IgG responses. A vaccination schedule of two immunizations separated by14 days was administered to C57BL/6J female mice (Figure 8A). After two immunizations, L_15_K_6_+PADRE‐J8 (20+37 µg/mouse/immunization) outperformed L_15_‐PADRE‐J8 (100 µg) in eliciting J8‐specific IgG. Significantly higher J8‐specific IgG titers were induced by L_15_K_6_+PADRE‐J8, despite a higher dose of L_15_‐PADRE‐J8 being administered following previous practices [9] (Figure 8B). This finding demonstrated that mixing a polyleucine derivative with an antigen may improve efficiency and the dose‐sparing effect compared to the conjugated equivalent. Similarly, antibodies from mice immunized with L_15_K_6_+PADRE‐J8 exhibited better antibacterial activity against D3840 and GC2 203 GAS strains than mice immunized with L_15_‐PADRE‐J8 (Figure 8C). Cytotoxicity of L_15_K_6_ and L_15_‐PADRE‐J8 were also evaluated in human cell lines, SW620 and HEK (Figure 8D). No significant cytotoxicity was observed in HEK cells in response to L_15_K_6_ or L_15_‐PADRE‐J8; however, L_15_‐PADRE‐J8 impacted viability of SW620 cells, indicating colorectal cytotoxicity.

### 2.9 L_15_K_6_ Enhanced the Immune Responses Induced by a GAS‐Derived Artificial Protein

**FIGURE 9:**
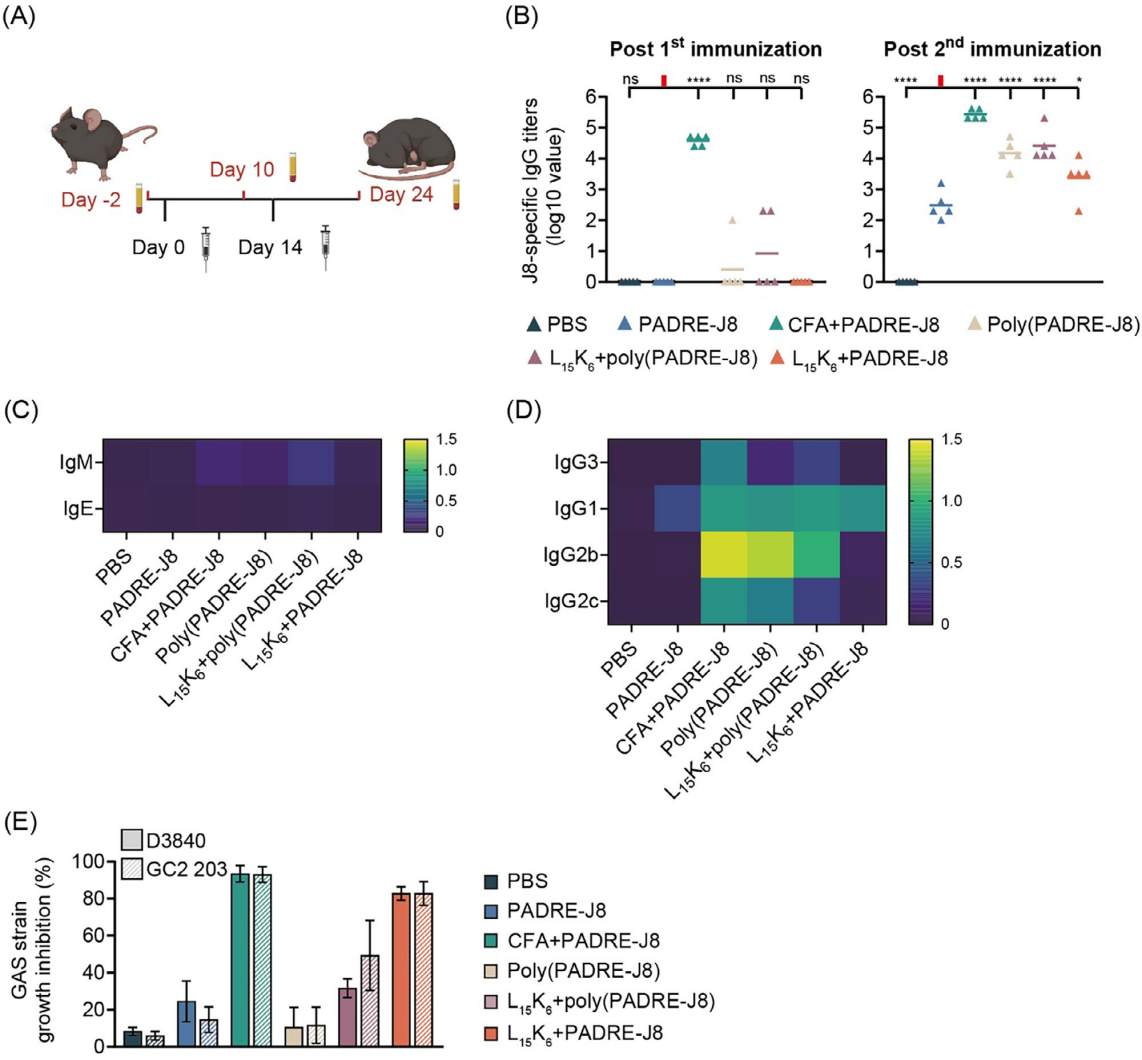
L_15_K_6_ enhanced murine immune responses against a GAS‐derived artificial protein. (A) Vaccination schedule administered to C57BL/6J female mice; (B) J8‐specific total IgG titers post first and second immunization; (C) Level of J8‐speciffic IgM and IgE (shown as mean absorbance, OD450 – OD540, n = 5); (D) Level of J8‐specific IgG subclass (IgG3, IgG1, IgG2b and IgG2c) profiling using pooled sera from each treatment group (performed in triplicates, shown as mean absorbance, OD450 – OD540, n = 3); (E) Bactericidal assay evaluating antibacterial potency of pooled sera from each group of immunized mice against clinically isolated GAS bacteria D3840 and GC2 203 (performed in duplicate, shown as percentage of bacterial growth inhibition). Data shown as mean ± SD. ns: not significant, *p* > 0.05; *: 0.01 < *p* ≤ 0.05; **:0.001 < *p* ≤ 0.01; ***: 0.0001 < *p* ≤ 0.001; ****: *p* ≤ 0.001.

In the previous experiment, L_15_K_6_ produced superior immune stimulating effects when co‐delivered with PADRE‐J8, despite both formulations being positively charged in aqueous solution. Given that PADRE‐J8 exhibited strong immunogenicity when delivered with L_15_K_6_, it was hypothesized that an artificial protein composed of multiple copies of PADRE‐J8 (i.e., Poly(PADRE‐J8)) would display enhanced immunogenicity when delivered with L_15_K_6_, compared to PADRE‐J8 alone. More alpha helices were present in the secondary structure of Poly(PADRE‐J8) (34%) than PADRE‐J8 (31%), and mixing L_15_K_6_ with poly(PADRE‐J8) produced even more alpha helices (45%) (Figure S5). To explore the immunogenicity of these formulations in vivo, C57BL/6J female mice were immunized twice, separated by 14 days (Figure 9A). Following the second immunization, poly(PADRE‐J8) strongly induced the production of J8‐specifc IgG, surpassing the levels triggered by PADRE‐J8 or L_15_K_6_+PADRE‐J8. However, contrary to the hypothesis, co‐delivery of L_15_K_6_ with poly(PADRE‐J8) did not increase J8‐specific IgG production compared to poly(PADRE‐J8) alone (Figure 9B).

Detectable levels of J8‐specific IgM were observed in several mice, particularly those immunized with CFA+PADRE‐J8, or L_15_K_6_+poly(PADRE‐J8) (Figure 9C). Given the short half‐life of IgM, these findings indicate good antigen retention and slow release at the injection site. No J8‐specific IgE was detected in any treatment group, indicating vaccine safety without Type I Hypersensitivity (Figure 9C). Finally, no differences were observed in C3a levels between groups at the time of blood collection, suggesting the absence of excessive ongoing inflammation (Figures S6 and S10). Subclasses of J8‐specific IgG (i.e., IgG3, IgG1, IgG2b and IgG2c) were profiled by ELISA using pooled sera (Figure 9D). Mice immunized with CFA+PADRE‐J8, poly(PADRE‐J8), or L_15_K_6_+poly(PADRE‐J8), displayed similar profiles of J8‐specific IgG1, IgG2b and IgG2c, while J8‐specific IgG1 represented the predominant IgG subclass in mice immunized with PADRE‐J8 or L_15_K_6_+PADRE‐J8 (Figure 9D). Interestingly, mice immunized with poly(PADRE‐J8) showed higher IgG2c but lower IgG3 levels than mice immunized with L_15_K_6_+poly(PADRE‐J8).

A bactericidal assay was performed to assess the quality of antibodies generated against different antigen types (Figure 9E). Despite high titers of J8‐specific IgG induced by poly(PADRE‐J8), these antibodies did not confer antibacterial activity against GAS strains. In contrast, antibodies induced by L_15_K_6_+poly(PADRE‐J8) produced higher antibacterial activity than antibodies induced by poly(PADRE‐J8), although the antibacterial activity was not high. Notably, antibodies induced by L_15_K_6_+PADRE‐J8 produced strong antibacterial activity, despite low titers after only two immunizations. Comparable antibacterial activity was observed between antibodies induced by CFA+PADRE‐J8 and L_15_K_6_+PADRE‐J8.

### 2.10 Positively Charged Polyleucine Derivative L_15_K_6_ Prompted the Immune Response Mounted by Protein Antigens

Immunopotentiation of amphiphilic polyleucine derivative, L_15_K_6,_ was subsequently confirmed with two model protein antigens, OVA and BSA. Positively charged L_15_K_6_ potentially interacts with negatively charged OVA or BSA through electrostatic interactions (Table S1).

**FIGURE 10:**
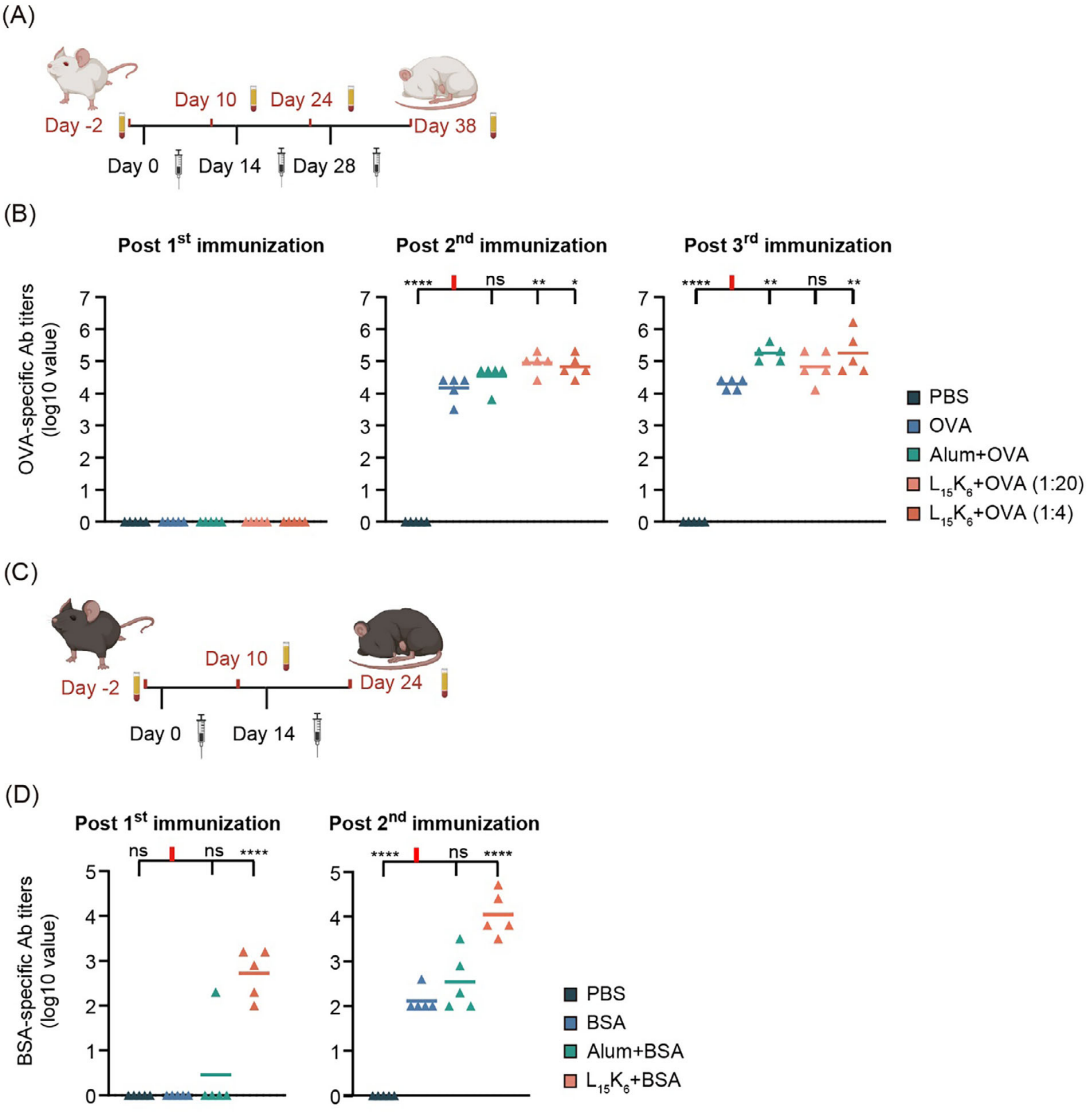
Mixing L_15_K_6_ with protein antigens enhanced antigen‐specific immune responses. (A) Immunization schedule with ovalbumin (OVA) antigen. BALB/c female mice were immunized three times separated by intervals of 14 days. Sera samples were collected 10 days post each vaccination; (B) OVA‐specific IgG titers in immunized mice post each vaccination. No OVA‐specific IgG response; (C) Immunization schedule with bovine serum albumin (BSA) antigen. C57BL/6J female mice were immunized twice with an interval of 14 days. Sera samples were collected 10 days post each vaccination; (D) BSA‐specific IgG titers in immunized mice post the primary and second immunization. ns: not significant, *p* > 0.05; *: 0.01 < *p* ≤ 0.05; **:0.001 < *p* ≤ 0.01; ***: 0.0001 < *p* ≤ 0.001; ****: *p* ≤ 0.001.

Two different doses of L_15_K_6_ (1 or 5 µg/mouse/immunization) administered three times were tested in combination with OVA in BALB/c mice (Figure 9A). No OVA‐specific IgG was detected after the first immunization in any group (Figure 9B). Following the second immunization, every formulation except for PBS, induced high anti‐OVA IgG titers. Mice that received OVA co‐delivered with L_15_K_6_ at two different doses showed higher OVA‐specific IgG than mice that received OVA alone (Figure 10B). After three immunizations, L_15_K_6_ (5 µg) co‐delivered with OVA generated comparable OVA‐specific IgG titers to aluminum salts co‐delivered with OVA. Both formulations induced significantly higher levels of OVA‐specific IgG production than OVA alone.

Subsequently, the L_15_K_6_ dose was increased to 20 µg/mouse/immunization and administered to C57BL/6J mice twice using BSA as the antigen (Figure 9C). Even with only a single immunization, L_15_K_6_+BSA induced the production of BSA‐specific IgG greater than BSA alone or aluminum co‐delivered with BSA (Figure 9D). After the second immunization, the highest BSA‐specific IgG titers were produced by mice that received L_15_K_6_+BSA. In contrast, after the second immunization of alum+BSA, mice only showed a similar IgG titer to mice that received a single immunization of L_15_K_6_+BSA.

## 3 Discussion

Commercially available immunopotentiators that promote strong immune responses are limited, making access to safe and effective immunopotentiators an urgent priority for the development of vaccines and other immunotherapeutics. Most immunopotentiators in approved vaccines are limited to aluminum salts or are emulsion‐based, while immunopotentiators under development range from polymers to proteins, lipids, metals, and metalloid molecules [28, 29, 30, 31, 32, 33].

Polyleucine‐antigen conjugates, such as L_15_‐PADRE‐J8 [9], L_10_‐8Qmin [11], and L_10_‐p3 [10]_,_ elicit robust antigen‐specific immune responses in mice; however, these formulations are laborious to synthesize and purify. In the current study, an amphiphilic polyleucine derivative was developed to waive the need for conjugation. Six hydrophilic amino acid residues were incorporated in the structure as a solubilizing moiety. This design was inspired by widely studied TLR2 agonist, Pam_2_CSK_4_, where four lysine residues were incorporated to improve solubility. Previous studies also showed that polyleucine derivatives containing 15 leucine residues (where the number of leucine residues was optimized [8, 9]) required more than four charged hydrophilic amino acids to ensure water solubility. Negatively, positively, and neutrally charged polyleucine derivatives (at a neutral pH condition) were obtained by modifying the composition of solubilizing moiety with glutamic acid or lysine.

Different to a polyleucine‐antigen conjugate, L_15_‐PADRE‐J8, physical mixture of polyleucine derivative and PADRE‐J8 (i.e., L_15_K_6_+PADRE‐J8) waived the need for time‐consuming and labor‐intensive synthesis and purification, achieving dose‐sparing effect and stronger immune regulation. In the present study, all three derivatives enhanced the uptake of peptide antigen PADRE‐J8 by DCs, although the most efficient antigen delivery was achieved by the negatively charged derivative L_15_E_6_. This was most likely due to complexes formed through electrostatic interactions between oppositely charged L_15_E_6_ particles and PADRE‐J8 antigens. Negatively charged L_15_E_6_ particles may also interact less with negatively charged proteins that are abundant in cell culturing media. However, high cellular uptake was not the main determinant of immune system activation, as the cationic polyleucine derivative L_15_K_6_ was the most potent activator of DCs, and it promoted antigen‐specific IgG production regardless of peptide or protein antigens (BSA or OVA).

Based on previous reports of only a few peptides being able to interact with TLR4 similarly to LPS [34] and prior research showing how synthetic polymers interact with TLR4 to agonize (negatively charged polymers) or antagonize (non‐cationic polymers) its activity, or indirectly influence TLR4 via receptor scavenging (polycationic polymers) [35], we hypothesized that polyleucine derivatives might also be TLR4 agonists. Here, positively charged L_15_K_6_ upregulated TLR4 expression in DCs more than L_15_E_6_ and L_15_(EK)_3_. After exposure to L_15_K_6_, increased levels of TNF‐α, but not IL‐6, were secreted by DCs, while LPS triggered significant secretion of both cytokines. In line with this, NF‐κB p65 was upregulated in DC2.4 after co‐culturing with L_15_K_6_ (Figure S8). Bulk RNA sequencing of THP‐1 cells after exposure to L_15_K_6_ also showed enrichment in TLR and NF‐κB signaling, suggesting polyleucine's possible interactions with TLRs. Taken together, the upregulation of TLR4 expression, increased NF‐κB p65 levels, and enrichment of TLR/NF‐κB signaling pathways across dendritic cells and THP‐1 cells supported a model in which L_15_K_6_ engages TLR‐associated NF‐κB signaling. While direct receptor binding was not assessed in this study, the concordant activation of these pathways across innate cell types have suggested a role for TLR‐mediated signaling in L_15_K_6_‐driven immune activation.

Antibodies produced by mice immunized with L_15_K_6_+PADRE‐J8 inhibited bacterial growth in vitro, while antibodies from mice that received L_15_E_6_+PADRE‐J8 or L_15_(EK)_3_+PADRE‐J8 were not highly antibacterial. This outcome was associated with the level of antibodies produced and possibly the ability of L_15_K_6_ to retain α‐helical secondary structures of J8 more effectively than L_15_E_6_ or L_15_(EK)_3_, enabling prompt B‐cell receptor recognition of the antigenic part of J8. Interestingly, J8‐specific IgG1 was the major IgG subclass in mice immunized with L_15_K_6_ co‐delivered with PADRE‐J8, while J8‐specific IgG2b was the major IgG subclass in mice immunized with CFA emulsified PADRE‐J8 or poly(PADRE‐J8). Consistent with other studies, this result indicates that murine IgG1 can also have decent antibacterial activity [36, 37]. Even though the highly immunogenic artificial protein poly(PADRE‐J8) failed to induce the production of antibodies that confer antibacterial activity, L_15_K_6_ still enhanced the quality of antibodies generated against poly(PADRE‐J8). This outcome was most likely due to conformational changes in poly(PADRE‐J8) that were detected by CD spectra. In future research, poly(PADRE‐J8) should be designed to maintain the conformational integrity of B‐cell epitopes to generate antibodies against the conformational entity within PADRE‐J8.

The immune responses induced by L_15_K_6_ were characterized primarily by robust antigen‐specific antibody production, with IgG1 representing the dominant subclass. This profile is consistent with a predominantly humoral‐biased response, which is appropriate for protection against extracellular bacterial pathogens such as Group A Streptococcus. Although cellular immune responses were not directly assessed in this study as humoral responses are more protective against GAS, the observed antibody subclass distribution suggests that L_15_K_6_ does not strongly skew toward a Th1‐dominant phenotype. However, our previous study on L_15_ conjugated to a cancer antigen showed that the conjugate could induce robust CD8 T cell responses and eradicate HPV‐related tumor [11]. Nevertheless, future studies examining T cell responses will be important to further define the balance between humoral and cellular immunity induced by L_15_K_6_.

Compared to other polymer‐based immunopotentiators, L_15_K_6_ exhibited several advantages. First, L_15_K_6_ is composed of naturally derived amino acids that are biodegradable, unlike other polymers, like polyethyleneimine, that are not inherently biocompatible and may cause cytotoxicity [38]. Second, L_15_K_6_ has a defined number of polymerized units – 15‐mer leucine residues, while other polymer materials can be highly heterogeneous, creating batch‐to‐batch variations. Lastly, L_15_K_6_ can be easily synthesized through standard solid‐phase peptide synthesis and self‐assembled into nanoparticles in aqueous solutions, while polymer‐based adjuvants, such as poly(methyl methacrylate), polyacrylamide, and poly(alkyl cyanoacrylates), require more complicated synthesis procedures, such as emulsion polymerization [39].

Although L_15_K_6_‐based formulations exhibited heterogeneity as assessed by TEM and DLS, such polydispersity is commonly observed in previously reported polyleucine derivatives [9]. Importantly, heterogeneous particle populations spanning from nano to micron scales may facilitate antigen uptake through various endocytic pathways, as particles of different sizes can be internalized by APCs via mechanisms such as phagocytosis, macropinocytosis, and clathrin‐ or caveolae‐mediated endocytosis [40]. In addition, particle charge and morphology are also known to play a role in mediating the uptake of nanoparticles by APCs. Consistent with this, despite the variability in particle size, L_15_K_6_ co‐delivered antigen nanocomplexes showed robust uptake by APCs in our study.

To date, peptide‐based immunopotentiators remain very limited; however, other peptide‐based immunopotentiators such as FK‐13 (FKRIVQRIKDFLR), an antimicrobial membrane disrupting peptide (LL‐37)‐derived peptide, have been reported to enhance anti‐tumor immune responses [41]. Like L_15_K_6_, FK‐13 can enhance antigen uptake by APCs and modulate the activation of APCs such as DCs [42]. However, L_15_K_6_ is effective as an independent adjuvant, while FK‐13 needs to be co‐adjuvanted with L‐phenylalanine‐based poly(ester amide) to trigger desired immune responses.

## 4 Conclusions

To assess the potential of polyleucine derivatives as immunopotentiators, this study synthesized polyleucine derivatives with three different solubilizing moieties. Among these derivatives, L_15_K_6_ emerged as a promising candidate due to its ability to significantly enhance antigen‐specific humoral responses. Like its counterparts, the amphiphilic nature of L_15_K_6_ facilitates self‐assembly into particles and recognition by immune cells. The positive charge of L_15_K_6_ likely enhances interactions with negatively charged cell membranes, promoting antigen uptake by APCs. Additionally, L_15_K_6_ can modulate TLR such as TLR2 and TLR4 expression in APCs, activating innate immune responses and enhancing receptor‐mediated endocytosis. This study demonstrated that when co‐delivered with various antigens of different charges and sizes, L_15_K_6_ outperformed traditional adjuvants, such as alum salts, by generating higher titers of antigen‐specific IgG. Notably, mixing L_15_K_6_ with an antigen was more immunogenic and dose‐sparing than the covalently conjugated counterpart, L_15_‐PADRE‐J8. When delivered with the artificial protein poly(PADRE‐J8), L_15_K_6_, improved the quality of antibodies generated against J8 as higher antibacterial activity was observed in response to antibodies induced by L_15_K_6_+poly(PADRE‐J8) than those induced by poly(PADRE‐J8) alone. These findings highlight the potential of L_15_K_6_ in subunit vaccine formulations.

## 5 Materials and Methods

### 5.1 Peptide Synthesis and Purification

All peptides used in this study were synthesized using tert‐butyloxycarbonyl (Boc) solid‐phase peptide synthesis [9, 43]. Boc‐protected l‐amino acids were assembled on p‐methyl‐benzhydryl‐amine hydrochloride (pMBHA·HCl) resin at 0.2 mmole scale, following the previously reported method [9, 43].

Briefly, the resin (0.2 mmole) was weighed and swelled in *N,N*‐dimethylformamide (DMF) and N,N‐diisopropylethylamine (DIPEA) (6.2 eq.) overnight. The coupling cycle for Boc synthesis included the following four steps:

- The Boc amino acids (0.84 mmole, 4.2 eq.) were activated by dissolving in 0.5 M hexafluorophosphate azabenzotriazole tetramethyl uranium (HATU) (1.6 mL, 4.0 eq.) in DMF followed by addition of DIPEA (0.26 mL, 6.2 eq.), and coupling twice (10 and 20 min).
- DMF wash five times.
- Deprotection of the Boc protecting group using TFA (2×1 min).
- DMF wash five times with the solvent aspirated between each wash. These four steps were repeated until the desired peptide sequence was obtained.

For deprotection of the Boc group from Boc‐Gln (Xan)‐OH, dichloromethane (DCM) was used to wash between the two trifluoroacetic acid (TFA) deprotection steps to avoid glutamine cyclization. Acetylation was performed after the last amino acid was coupled using acetylation solution (2.5% DIPEA and 2.5% acetic anhydride in DMF). The formyl group from tryptophan was removed using 20% piperidine in DMF solution (5 and 10 min). The resin was then washed with DMF (3X), followed by DCM (3X), and methanol (1X) before transferring the resin‐peptide to a desiccator overnight.

Peptides were cleaved from the resin using anhydrous hydrogen fluoride (HF) with p‐cresol or p‐thiocresol as scavengers [44]. Following evaporation of HF, cleaved peptides were washed with cold diethyl ether or a mixture of diethyl ether and n‐hexane (1:1). Precipitated compounds were dissolved in mixture of solvent A (100% Milli‐Q water containing 0.1% TFA) and solvent B (90% acetonitrile and 10% Milli‐Q water containing 0.1% TFA) depending on the hydrophobicity of the peptide. After filtration, the peptide was purified using a Shimadzu preparative reversed‐phase HPLC (RP‐HPLC; Kyoto, Japan) instrument (LC‐20AP x 2, CBM‐20A, SPD‐20A, FRC‐10A) with a 20.0 mL/min flow rate on a C18 (218TP1022; 10 µm, 22 × 250 mm) or C4 (214TP1022; 10 µm, 22 × 250 mm) column depending on the hydrophobicity of the compound. Once purified, peptide purity was determined using an analytical RP‐HPLC on a C18 (218TP54; 5 µm,4.6 × 250 mm) or C4 (214TP54; 5 µm, 4.6 × 250 mm) Vydac column, with a 0–100% gradient of solvent B for 40 min at 214 nm. ESI‐MS was performed on a LCMS‐2020 Shimadzu (Kyoto, Japan) instrument (DGU‐20A3, LC‐20Ad x 2, SIL‐20AHT, STO‐20A) and Analyst 1.4 software (Applied Biosystems/MDS Sciex, Toronto, Canada) (Perkin‐Elmer‐Sciex API3000) to validate molecular weight of the compound.

J8 (QAEDKVKQSREAKKQVEKALKQLEDKVQ). Yield: 23%. Molecular weight: 3323.81 g/mol. ESI‐MS [M + 3H]^3+^ m/z 1109.4 (calc. 1108.9), ESI‐MS [M + 4H]^4+^ m/z 832.1 (calc. 832.0), [M + 5H]^5+^ m/z 666.0 (calc. 665.8), [M + 6H]^6+^ m/z 555.2 (calc. 555.0). t_R_ = 21.4 min (0–100% solvent B; C18 column); purity ≥99%.

PADRE‐J8 (AFKVAAWTLKAAA‐QAEDKVKQSREAKKQVEKALKQLEDKVQ). Yield: 30%. Molecular weight: 4653.42 g/mol. ESI‐MS [M + 3H]^3+^ m/z 1552.7 (calc. 1552.1), ESI‐MS [M + 4H]^4+^ m/z 1164.5 (calc. 1164.4), [M + 5H]^5+^ m/z 932.1 (calc. 931.7), [M + 6H]^6+^ m/z 776.8 (calc. 776.6), [M + 7H]^7+^ m/z 666.0 (calc. 665.8). t_R_ = 24.5 min (0–100% solvent B; C18 column); purity ≥99%.

L_15_K_6_ (LLLLLLLLLLLLLLLKKKKKK). Yield: 10%. Molecular weight: 2525.52 g/mol. ESI‐MS [M + 2H]^2+^ m/z 1263.8 (calc. 1263.8), ESI‐MS [M + 3H]^3+^ m/z 843.4 (calc. 842.8), [M + 4H]^4+^ m/z 632.8 (calc. 632.4). t_R_ = 36.4 min (0–100% solvent B; C4 column); purity ≥99%.

L_15_E_6_ (LLLLLLLLLLLLLLLEEEEEE). Yield: 8%. Molecular weight: 2531.16 g/mol. ESI‐MS [M + H]^1+^ m/z 2532.5 (calc. 2532.2), ESI‐MS [M + 2H]^2+^ m/z 1267.2 (calc. 1266.6), [M + 3H]^3+^ m/z 844.9 (calc. 844.7). t_R_ = 43.6 min (0–100% solvent B; C4 column); purity ≥99%.

L_15_(EK)_3_ (LLLLLLLLLLLLLLLEKEKEK). Yield: 13%. Molecular weight: 2528.34 g/mol.

ESI‐MS [M + H]^1+^ m/z 2528.2 (calc. 2529.34), ESI‐MS [M + 2H]^2+^ m/z 1264.9 (calc. 1265.2), [M + 3H]^3+^ m/z 843.7 (calc. 843.8). t_R_ = 40.4 min (0–100% solvent B; C4 column); purity ≥99%.

### 5.2 Vaccine Formulation

Vaccine candidates (L_15_K_6_+PADRE‐J8, L_15_(EK)_3_+PADRE‐J8, L_15_E_6_+PADRE‐J8, L_15_K_6_+BSA, or L_15_K_6_+OVA) were formulated by physically mixing stock solutions of antigens and immunopotentiators at working concentrations. To prepare stock solutions of immunopotentiators, polyleucine derivatives (L_15_K_6_, L_15_(EK)_3_ or L_15_E_6_) were solubilized in sterile irrigation water before being sonicated and mixed by vortex. Next, 10X PBS was added to produce 1X PBS osmolarity. All antigens were easily solubilized in 1X PBS.

### 5.3 Dynamic Light Scattering and Transmission Electron Microscopy

The particle size, polydispersity index (PDI), and surface charge (zeta potential) of all vaccine components or vaccine formulations were assessed in disposable folded capillary zeta cells (DTS1070) at a concentration of 0.2 mg/mL, using a Malvern Zetasizer Nano ZS (Malvern Instruments Ltd., Worcestershire, United Kingdom), at 25°C with a 173° back‐scattering angle. Five measurements (11 runs per measurement) were recorded for each sample. A HT7700 Exalens instrument (HITACHI Ltd., Tokyo, Japan) was used to visualize morphology of nanoparticles and aggregates formed by vaccine components and vaccine formulations dissolved in water. Briefly, 50 µL of vaccine components or vaccine formulations (0.2 mg/mL) was loaded on a glow‐discharged carbon‐coated grind and incubated for 2 min before particle settling. Filter paper was used to remove excess liquid before particles were stained with either 2% uranyl acetate (for negatively charged particles) or phosphotungstic acid (for positively charged particles) for 2 min. Lastly, the grind was air‐dried before imaging.

### 5.4 Circular Dichroism Spectroscopy

The secondary structure of polyleucine derivatives or physical mixtures of polyleucine derivatives and peptide antigen PADRE‐J8 (0.2 mg/mL or less) was analyzed by CD spectroscopy. Measurement parameters were as follows: 5 nm bandwidth, 50 nm/min scan rate, 2s response time, and 1 nm intervals over the wavelength range of 195–260 nm. Measurements were performed at 25°C in a nitrogen atmosphere. Data are reported as the mean of six measurements. Mean residue molar ellipticity (deg × cm^2^ × dmol^−1^) was calculated using the formula [θ] = mdeg/(l × c × n). l = path length (1 mm); c = peptide concentration (M); n = the number of residues in the peptide.

### 5.5 Cell Culturing

DC2.4 cells (SCC142) were cultured in RPMI‐1640 medium supplemented with 10% v/v fetal bovine serum (FBS), 2.5% v/v HEPES (1 M) buffer, 1% v/v L‐glutamine, 1% v/v MEM NEAA (100X), 1% v/v PSG (100X), and 0.00054% v/v 2‐mercaptoethanol in a T75 or T25 flask. Culture media was refreshed every 24 h until ∼80% cell confluency. For cell collection, media was removed via pipetting and cells were washed once with 1X PBS. Approximately 5–7 mL of trypsin‐EDTA was added to culture flasks and cells were incubated for 5 min at 37°C. The flasks were gently rocked or tapped before the addition of culture media. Cell suspensions were then centrifuged at 300 x g for 5 min at room temperature with cell pellets re‐suspended in culture media. THP‐1 cells were cultured in RPMI‐1640 medium supplemented with 10% v/v FBS, 1% v/v L‐glutamine and 1% v/v PSG (100X). One million (1 × 10^6^) cells plated in U‐bottom 96 well plate and stimulated with 10 µM of antigen for 24 h and cells were lysed for RNA extraction. RNA was extracted per manufacturer's instructions (Qiagen, Hilden, Germany). RNA sequencing was performed at Genewiz from Azenta Life Sciences (Burlington, Massachusetts, United States).

BMDMs were generated as previously described [45]. Briefly, twenty‐five‐week‐old mice were sacrificed by CO_2_ asphyxiation. Femurs and tibias were extracted and flushed in DMEM for bone marrow isolation. Bone marrow (BM) cells from each bone were plated in a 15 cm petri dish containing 25 mL Macrophage Differentiation Medium (20% L cell‐conditioned medium, 10% fetal bovine serum, 2 mM L‐glutamine, 1 mM sodium pyruvate, and 10 mM HEPES). 5 mL pre‐warmed Macrophage Differentiation Medium was added on days 2 and 4 post‐seeding. On day 6, medium was removed, adherent cells washed with 10 mL pre‐warmed PBS. Cells were detached by adding 7.5 mL ice‐cold sterile 1 mM EDTA/PBS followed by incubation for 10 min at 4°C. For harvesting, cells were washed off with ice‐cold sterile PBS twice and collected in a total volume of 25 mL PBS into a sterile 50 mL tube and centrifuged at 282 x g for 7 min, 4°C. Cell pellets were resuspended in 10 mL Macrophage Maintenance Medium (10% L cell‐conditioned medium, 10% fetal bovine serum, 2 mM L‐glutamine, 1 mM sodium pyruvate, and 10 mM HEPES). Cell concentration and viability were determined automatically using a TC20 Automated Cell Counter.

### 5.6 Dendritic Cell Uptake and Maturation

DC uptake and maturation assays were conducted as described previously with modifications [24]. Briefly, 1 × 10^5^ DC2.4 cells were seeded per well in 24‐well culture plates, treated with vaccine formulations, and incubated overnight in 5% CO_2_ at 37°C.

In the uptake assay, 2.5 µM fluorescence‐labeled antigen was added with or without polyleucine derivatives. Cells were then incubated for 4 h prior to collection. Live/dead staining and cell fixation were performed before flow cytometry analysis of fluorescence. For confocal microscopy, coverslips were added to each well of the 24‐well plates prior to seeding cells. Cells on coverslips were then washed, fixed using 4% paraformaldehyde, and stained with DAPI and wheat germ agglutinin to visualize the nucleus and cell membrane. Coverslips were mounted and observed by a FV3000 Confocal Laser Scanning Microscope (Olympus, Shinjuku City, Japan).

In the maturation assay, 10 µM of antigen with or without polyleucine derivatives was added. Cells were incubated for 24 h prior to collection, followed by FcR blocking, live/dead staining, antibody staining, and fixation. Maturation marker expression was also analyzed by flow cytometry.

### 5.7 Cytokine Profiling

Cytokines were profiled in DC2.4 culture supernatants by ELISA kits from Absin (Shanghai, China). Up to 1 × 10^5^ DC2.4 cells were seeded into each well of 24‐well culture plates for overnight incubation. Next, 10 µM of vaccine formulations were added to each well in duplicate for another 24 h incubation. Culture media was collected by centrifugation at 300 x g for 5 min. Cytokines were measured in supernatants from each treatment group via sandwich ELISA according to manufacturer's instructions (Absin, Shanghai, China).

### 5.8 Flow Cytometry Analysis

Flow cytometry events were acquired using a BD LSRFortessa X‐20 Cell Analyzer with BD FACSDiva software (BD Biosciences, Franklin Lakes, United States). Automatic compensation was conducted using compensation beads stained with single‐colored fluorescent‐conjugated antibodies. After the compensation set‐up, the events for fully stained samples and FMO samples were acquired. At least 10,000 events were recorded for each sample. Finally, the data were exported as FCS files and analyzed using FlowJo v10.8 software.

### 5.9 In Vivo Study

Animal studies were conducted following regulations by National Health and Medical Research Council (NHMRC) of Australia (Australian Code of Practice for the Care and Use of Animals for Scientific Purposes). All animal procedures and protocols were approved by the University of Queensland Animal Ethics Committee (AEC), AEC approval number: 2021/AE000311.

Female C57BL/6J mice, aged 4–6 weeks, were sorted into groups of five. Mice in each group were immunized subcutaneously three times, with intervals of 14 days. Vaccine formulations administered to each group are detailed in the tables (Tables S2–S6).

Blood samples were collected for baseline data two days prior to the primary immunization and 10 days after each immunization. Blood sampling was performed via tail vein bleeding (before and throughout the study) or cardiac puncture for the final sample. On the day of cardiac puncture, which occurred 10 days after the final immunization, mice were euthanized using CO_2_ before blood collection. Collected blood samples were centrifuged at 1,500 x g for 10 min at room temperature before sera were carefully extracted by pipetting, aliquoted, and stored at −80°C.

### 5.10 Enzyme‐Linked Immunosorbent Assay for Immunoglobulins

The J8‐specific IgG, IgE, IgG1, IgG2b, IgG2c, or IgG3 in murine sera, were measured by enzyme‐linked immunosorbent assays (ELISAs). Polycarbonate plates were prepared by coating each well with 100 µL J8 (0.5 mg/mL in carbonate coating buffer, pH 9.6) and incubating at 37°C for 90 min. After coating, plate contents were discarded before a blocking solution was added (i.e., 150 µL 5% skim milk dissolved in PBST buffer) to each well. Plates were then incubated at 4°C overnight before being washed three times each with water and PBST.

For IgG characterization, each serum sample was diluted in 1X PBS at a 1:100 ratio. 200 µL of diluted samples were then added to assigned wells of the plate before two‐fold serial dilutions in 0.5% skim milk in PBST. For IgE characterization, individual serum samples in each group were diluted at a 1:100 ratio without serial dilution. For IgG1, IgG2b, IgG2c, and IgG3 characterization, sera samples were pooled from each group and diluted at a 1:100 ratio without serial dilution.

Plates were incubated for 90 min at 37°C before being washed as described above. A detection antibody solution was prepared by diluting peroxidase‐conjugated goat anti‐mouse IgG at a ratio of 1:3000, IgE at a ratio of 1:1000, IgG1 at a ratio of 1:6000, IgG2b at a ratio of 1: 2000, IgG2c at a ratio of 1:10 000, or IgG3 at a ratio of 1:1000 using 0.5% skim milk in PBST. 100 µL of diluted solutions was added to each well and plates were incubated for another 90 min at 37°C.

Plate contents were discarded, and wells were washed. The chromogenic substrate for the peroxidase enzyme, o‐phenylenediamine dihydrochloride (OPD), was prepared by dissolving one 5 mg tablet in 20 mL of 1X Stable Peroxide Buffer, according to manufacturer's instructions. To start the colorimetric reaction, 100 µL substrate was added to each well before plates were incubated at room temperature in darkness for 20 min. To stop the reaction, 50 µL stop solution containing sulfuric acid was added to each well. The reaction was quantified by measuring absorbance at a wavelength of 450 nm.

The IgG titers were calculated using [mean+3*standard deviation] of the absorbance of two naïve serum samples as the cut‐off, with 0.07 serving as the additional cut‐off. For IgE, IgG1, IgG2b, IgG2c and IgG3, the absorbance was calibrated at a wavelength of 540 nm (A_450nm_‐A_540nm_).

### 5.11 Bactericidal Assessment

The bactericidal assay was conducted to determine if sera obtained from immunized mice could opsonize GAS as described previously [46, 47, 48]. Clinical bacterial isolates acquired from Princess Alexandra Hospital included: (i) GC2 203, obtained from a wound swab; and (ii) D3840, obtained from nasopharyngeal swabs.

Bacterial cultures were initiated by streaking onto Todd‐Hewitt broth (THB) agar plates supplemented with 5% yeast extract, followed by incubation at 37°C for 24 h. Subsequently, a single bacterial colony was inoculated into 5 mL of THB broth, also supplemented with 5% yeast extract, and incubated at 37°C for an additional 24 h. Bacteria were cultured until reaching a density of ∼4.6 × 10^6^ CFU/mL.

Bacterial cultures were then diluted 100‐fold in PBS. A mixture was prepared consisting of 10 µL of the diluted bacterial culture, 10 µL of heat‐inactivated serum (by incubation in a 50°C water bath for 30 min), and 80 µL of horse blood. This mixture was incubated at 37°C for 3 h in a 96‐well plate to facilitate interactions between bacteria and sera. After incubation, 10 µL of bacterial suspensions was spread onto THB agar plates supplemented with 5% yeast extract and 5% horse blood. Plates were then incubated at 37°C for an additional 24 h. Quantitative assessment of bacterial growth inhibition was performed by counting CFUs on the incubated agar plates. Assays were conducted in duplicates.

### 5.12 Statistical Analysis

Statistical analysis and graphical representations were produced using Prism version 10.2.1 (GraphPad). The data underwent one‐way ANOVA accompanied by Tukey's multiple comparison. A significance threshold of p<0.05 was adopted, unless otherwise specified.

## Conflicts of Interest

The authors declare no conflict of interest.

## Supporting information




**Supporting File**: smll72876‐sup‐0001‐SuppMat.docx.

## Data Availability

The data that support the findings of this study are available from the corresponding author upon reasonable request.
