# Supplementary material for: Development of a Novel Polyleucine‐Based Immunopotentiator for Subunit Vaccines Against Group A Streptococcus
Source: Small. 2026 Feb 17;22(22):e11459. doi: 10.1002/smll.202511459 (PMC13089101; doi:10.1002/smll.202511459)
Supplement: Supplementary file 1 — Supporting File: smll72876‐sup‐0001‐SuppMat.docx. [file SMLL-22-e11459-s001.docx]

Supporting Information

**Development of A Novel Polyleucine-Based Immunopotentiator for Subunit Vaccines against Group A Streptococcus**

*Lantian Lu^,*^, Jazmina L. Gonzalez Cruz, Rabina Giri, Yuexiao Zhang, Sahra Bashiri, Leslie C. Domínguez Cadena, Shuhang Li, Zeinab G. Khalil, Waleed M. Hussein, Jingwen Wang, Jolynn Kiong, Xuehan Lu, Jiahui Zhang, Miao Lu, Wenbin Huang, Wei Yang Kong, Ummey Jannatun Nahar, Prashamsa Koirala, Zhong Guo, Patricia Walden, Antje Blumenthal, Jakob Begun, Kirill Alexandrov, Istvan Toth, Rachel J. Stephenson, Mariusz Skwarczynski^*^*

L. Lu, Y. Zhang, S. Bashiri, J. Wang, J. Zhang, M. Lu, W. Huang, U. J. Nahar, P. Koirala, I. Toth, R. J. Stephenson, M. Skwarczynski

School of Chemistry and Molecular Biosciences, The University of Queensland, St Lucia, Brisbane, QLD 4072, Australia.

Email: [m.skwarczynski@uq.edu.au](mailto:m.skwarczynski@uq.edu.au); [lantlu@iu.edu](mailto:lantlu@iu.edu)

L. Lu, J. L. Gonzalez Cruz, S. Bashiri, L. C. *Domínguez Cadena,* X. Lu, J. Zhang, W. Y. Kong, A. Blumenthal

Frazer Institute, The University of Queensland, Translational Research Institute, Woolloongabba, Brisbane, QLD 4102, Australia.

R. Giri, S. Li, J. Begun

Mater Research Institute, Translational Research Institute, The University of Queensland, Woolloongabba, QLD, 4102, Australia.

Z. G. Khalil, J. Kiong

Australian Institute for Bioengineering and Nanotechnology, The University of Queensland, QLD, 4072, Australia.

Z. G. Khalil, W. M. Hussein, I. Toth

Institute for Molecular Bioscience, The University of Queensland, St Lucia, QLD 4072, Australia.

Z. Guo, P. Walden, K. Alexandrov

School of Biology and Environmental Science, Queensland University of Technology, Brisbane, QLD 4000, Australia.

Z.G. Khalil, I. Toth

School of Pharmacy, The University of Queensland, Woolloongabba, QLD 4102, Australia.

L. Lu

Department of Microbiology and Immunology, Indiana University School of Medicine, Indianapolis, IN 46202, United States.

**Supplementary Tables**

**Supplementary Table 1.** DLS characterization of polyleucine derivatives

| Formulation | Size (nm) | PDI | Charge (mV) |
| --- | --- | --- | --- |
| *Polyleucine derivative* | | | |
| L_15_K_6_ | Polydipersed size | 0.765 ± 0.326 | 38.9 ± 2.6 |
| L_15_(EK)_3_ | Polydipersed size | 0.76 ± 0.08 | 33.3 ± 4.4 |
| L_15_E_6_ | Polydipersed size | 0.95 ± 0.12 | -56.3 ± 1.3 |
| *Antigen* | | | |
| OVA | 43 ± 5  275 ± 14  5006 ± 136 | 0.34 ± 0.04 | -26.9 ± 3.0 |
| BSA | 7  276 ± 83  4307 ± 860 | 0.38 ± 0.12 | -23.7 ± 3.4 |
| Poly(PADRE-J8) | 374 ± 25  5121 ± 168 | 0.24 ± 0.03 | 11.7 ± 2.2 |
| PADRE-J8 | 370 ± 24  3366 ± 71 | 0.68 ± 0.10 | 30.3 ± 1.8 |
| *Polyleucine derivative + Ag mixture* | | | |
| L_15_K_6_ + OVA (1:20) | 238 ± 66  1175 ± 436  4803 ± 216 | 0.55 ± 0.11 | -18.3 ± 0.9 |
| L_15_K_6_ + OVA (5:20) | 534 ± 42  2665 ± 469  4965 ± 445 | 0.35 ± 0.05 | 25.4 ± 0.3 |
| L_15_K_6_ + BSA | Polydispersed size | 0.65 ± 0.13 | 29.8 ± 0.8 |
| L_15_K_6_ + poly(PADRE-J8) | Polydispersed size | 0.87 ± 0.11 | 18.0 ± 3.6 |
| L_15_K_6_ + PADRE-J8 | Polydispersed size | 0.79 ± 0.26 | 51.2 ± 1.2 |
| L_15_(EK)_3_ + PADRE-J8 | 130 ± 16  655 ± 145 | 0.80 ± 0.11 | 55.3 ± 2.2 |
| L_15_E_6_ + PADRE-J8 | 246  573 ± 61 | 0.98 ± 0.02 | 51.8 ± 1.5 |

**Supplementary Table 2.** Vaccine formulation for animal study 1 (Polyleucine derivatives adjuvanted GAS peptide vaccines)

| Group # | Immunopotentiator  (in PBS) | Immunopotentiator dose (per mouse) | Antigen  (In PBS) | Antigen dose (per mouse) |
| --- | --- | --- | --- | --- |
| 1 | - | - | - | - |
| 2 | - | - | PADRE-J8 | 37.0 μg |
| 3 | CFA | 25.0 μL | PADRE-J8 | 37.0 μg |
| 4 | L_15_K_6_ | 20.0 μg | PADRE-J8 | 37.0 μg |
| 5 | L_15_E_6_ | 20.0 μg | PADRE-J8 | 37.0 μg |
| 6 | L_15_(EK)_3_ | 20.0 μg | PADRE-J8 | 37.0 μg |

**Supplementary Table 3.** Vaccine formulation for animal study 2 (GAS peptide vaccines adjuvanted with polyleucine as a conjugate or a physical mixture)

| Group # | Immunopotentiator  (in PBS) | Immunopotentiator dose (per mouse) | Antigen  (in PBS) | Antigen dose (per mouse) |
| --- | --- | --- | --- | --- |
| 1 | - | - | - | - |
| 2 | CFA | 25.0 μL | PADRE-J8 | 37.0 μg |
| 3 | L_15_K_6_ | 20.0 μg | PADRE-J8 | 37.0 μg |
| 4 | **L_15_**-PADRE-J8* | ~26.7μg | L_15_-**PADRE-J8*** | 73.3 μg |

* Corresponding adjuvant or antigen in the conjugate was highlighted in red

**Supplementary Table 4.** Vaccine formulation for animal study 3 (GAS artificial protein vaccines vs. peptide vaccine)

| Group # | Immunopotentiator  (in PBS) | Immunopotentiator dose (per mouse) | Antigen  (in PBS) | Antigen dose (per mouse) |
| --- | --- | --- | --- | --- |
| 1 | - | - | - | - |
| 2 | - | - | PADRE-J8 | 37.0 μg |
| 3 | CFA | 25.0 μL | PADRE-J8 | 37.0 μg |
| 4 | L_15_K_6_ | 20.0 μg | PADRE-J8 | 37.0 μg |
| 5 | - | - | Poly(PADRE-J8) | 37.0 μg |
| 6 | L_15_K_6_ | 20.0 μg | Poly(PADRE-J8) | 37.0 μg |

**Supplementary Table 5.** Vaccine formulation for animal study 4 (OVA adjuvanted with polyleucine derivatives)

| Group # | Immunopotentiator  (in PBS) | Immunopotentiator dose (per mouse) | Antigen  (in PBS) | Antigen dose (per mouse) |
| --- | --- | --- | --- | --- |
| 1 | - | - | - | - |
| 2 | - | - | OVA | 20.0 μg |
| 3 | Imject^TM^ alum | 25.0 μL | OVA | 20.0 μg |
| 4 | L_15_K_6_ | 1.0 μg | OVA | 20.0 μg |
| 5 | L_15_K_6_ | 5.0μg | OVA | 20.0 μg |

**Supplementary Table 6.** Vaccine formulation for animal study 5 (BSA adjuvanted with L_15_K_6_)

| Group # | Immunopotentiator  (in PBS) | Immunopotentiator dose (per mouse) | Antigen  (in PBS) | Antigen dose (per mouse) |
| --- | --- | --- | --- | --- |
| 1 | - | - | - | - |
| 2 | - | - | BSA | 20.0 μg |
| 3 | Imject^TM^ alum | 25.0 μL | BSA | 20.0 μg |
| 4 | L_15_K_6_ | 20.0 μg | BSA | 20.0 μg |

**Supplementary Figures**


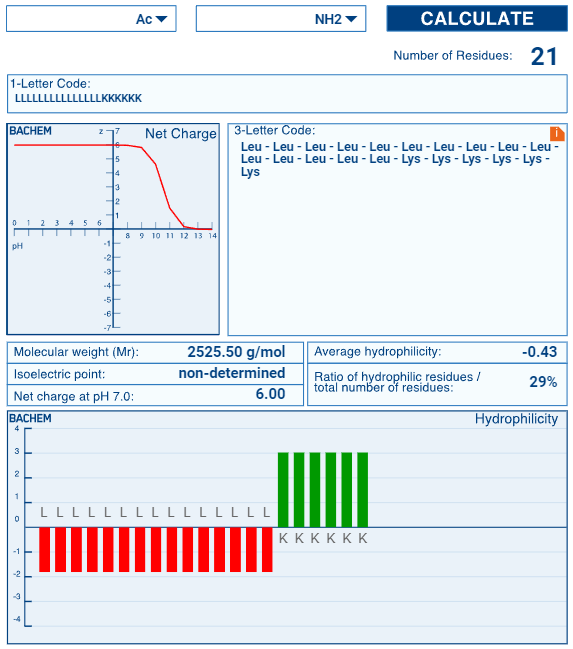

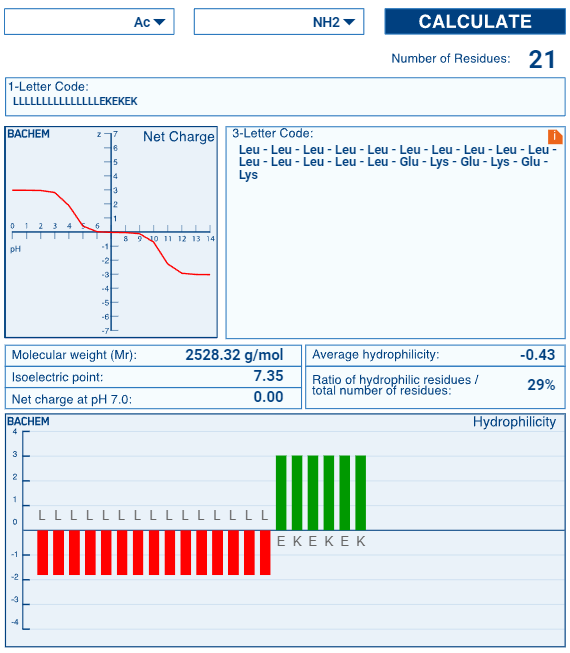

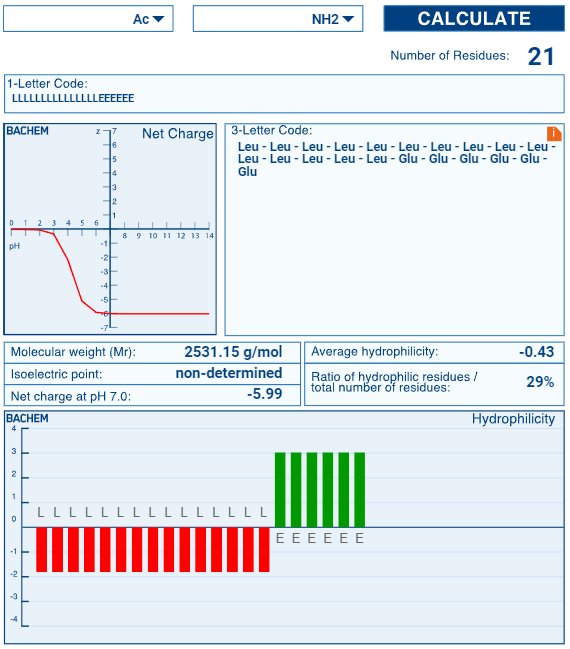


L_15_E_6_

L_15_(EK)_3_

L_15_K_6_

**Supplementary Figure 1.** Calculated net charge of polyleucine derivatives at pH 7.0. Figure derived from Bachem peptide calculator (<https://www.bachem.com/knowledge-center/peptide-calculator/>).

J8

t_R=21.4 min_

PADRE-J8

t_R=25.8 min_

L_15_K_6_

t_R=36.4 min_

L_15_(EK)_3_

t_R=40.4 min_

L_15_E_6_

t_R=43.6 min_

**Supplementary Figure 2.** MS and HPLC characterization of peptide antigens and polyleucine derivatives.

L_15_K_6__size distribution by number

L_15_K_6__size distribution by intensity


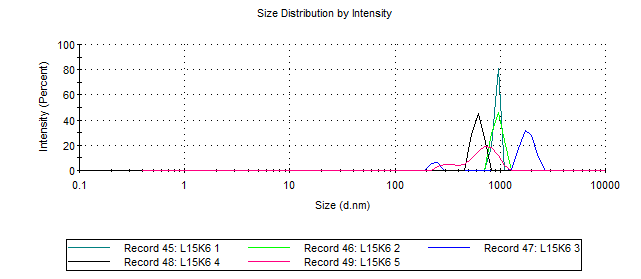

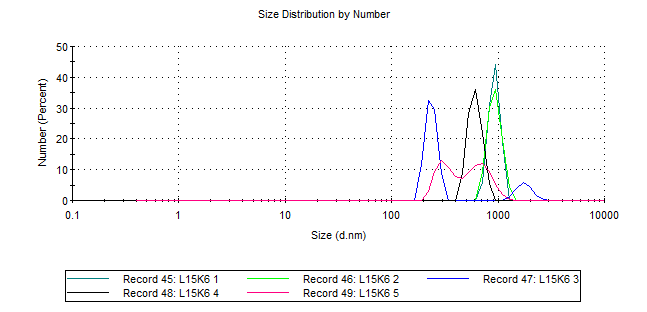


L_15_K_6__zeta potential


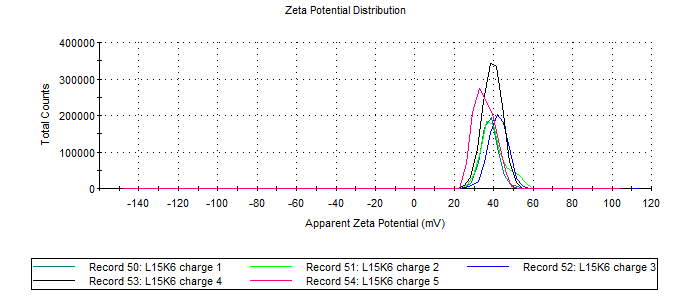


L_15_(EK)_3__size distribution by intensity

L_15_(EK)_3__size distribution by number


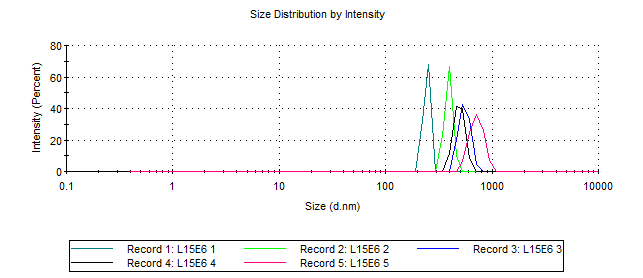

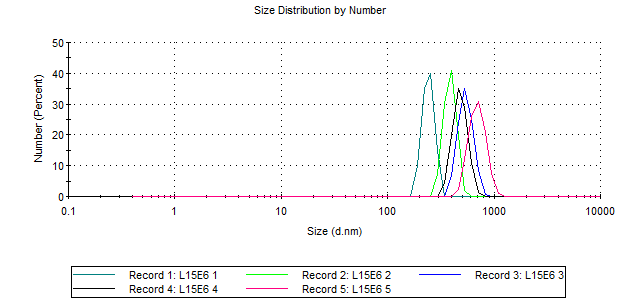

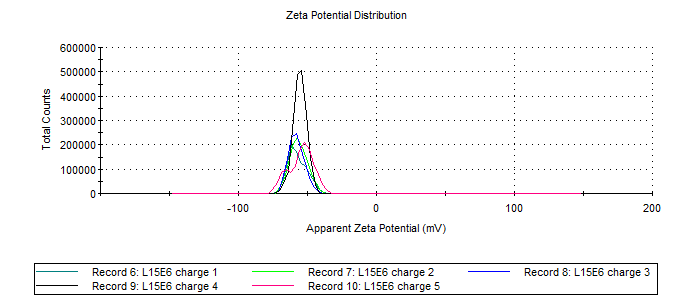


L_15_E_6__size distribution by intensity

L_15_E_6__size distribution by number

L_15_(EK)_3__zeta potential


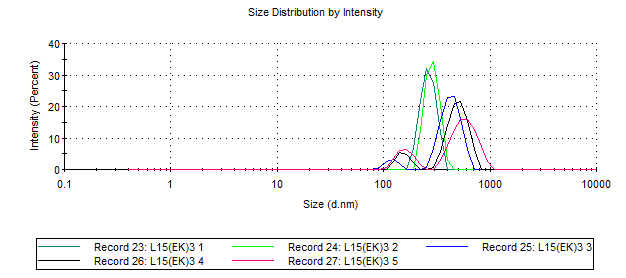

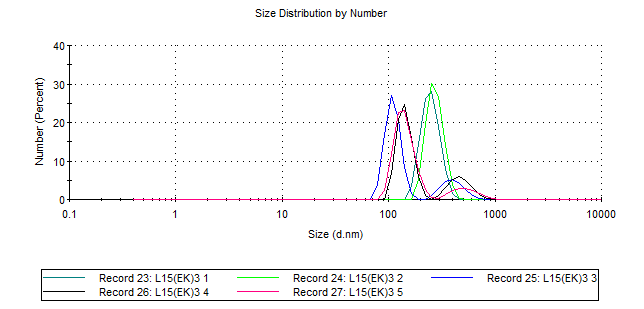


L_15_E_6__zeta potential


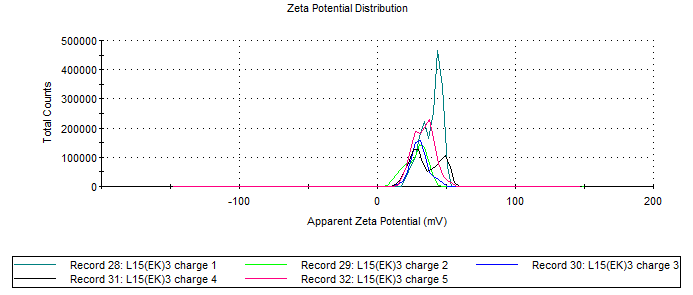


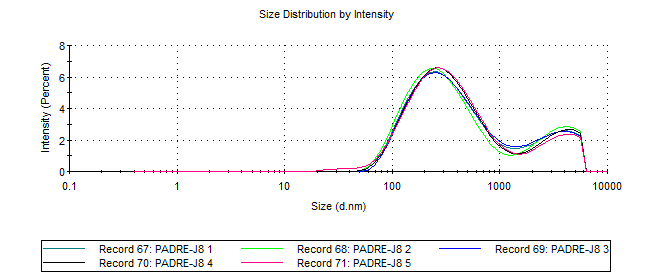

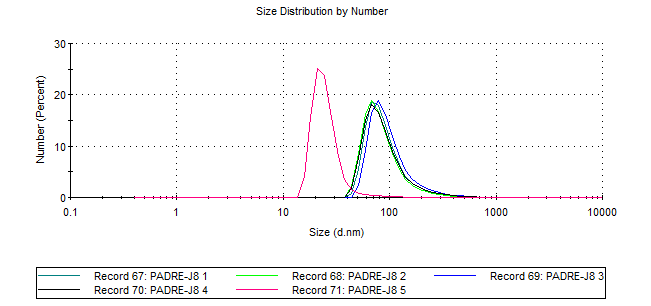


PADRE-J8_zeta potential

PADRE-J8_size distribution by number

PADRE-J8_size distribution by intensity


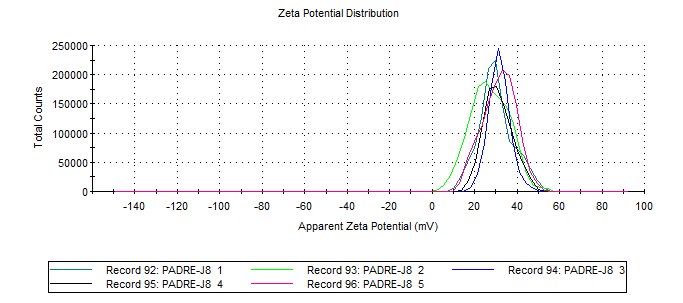


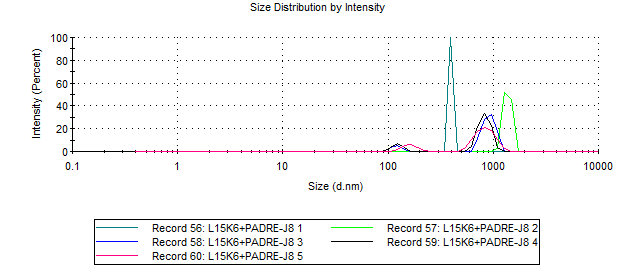

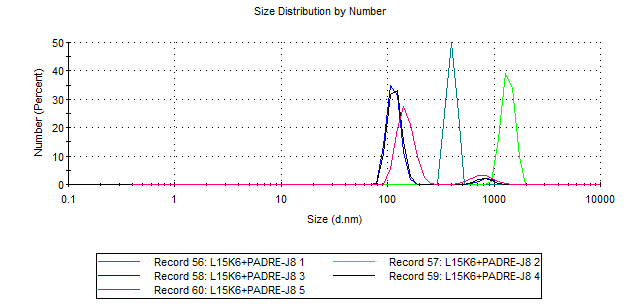


L_15_K_6_+PADRE-J8_zeta potential

L_15_K_6_+PADRE-J8_size distribution by number

L_15_K_6_+PADRE-J8_size distribution by intensity


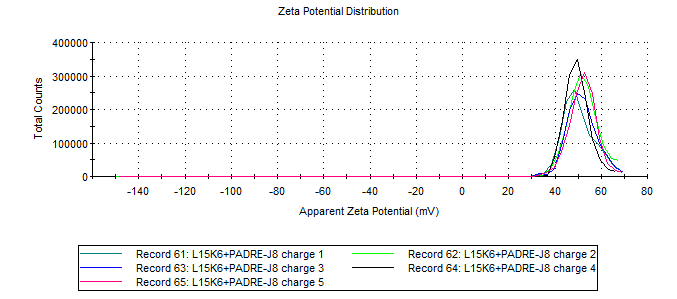


L_15_(EK)_3_+PADRE-J8_size distribution by number

L_15_(EK)_3_+PADRE-J8_size distribution by intensity


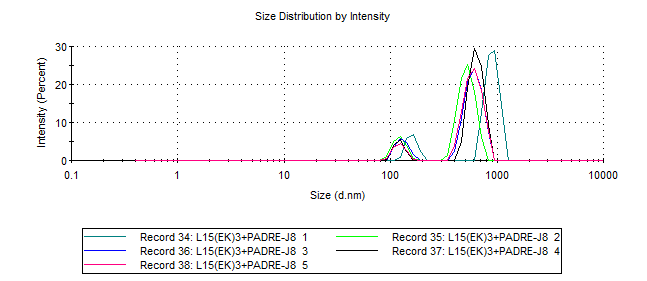

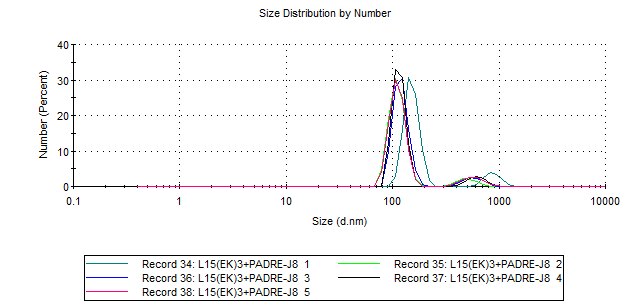


L_15_(EK)_3_+PADRE-J8_zeta potential


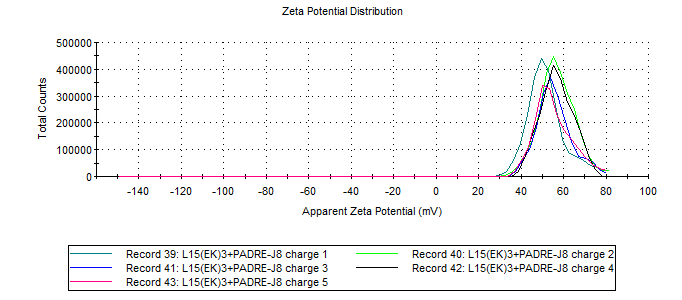


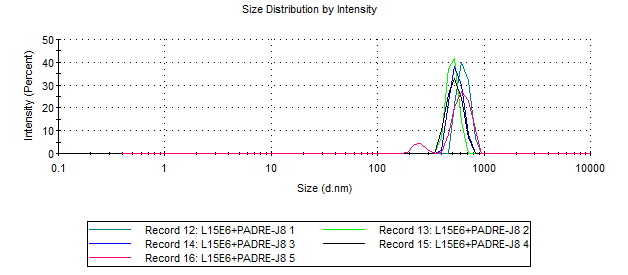

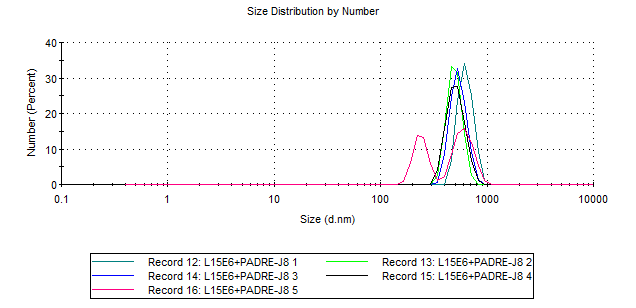


L_15_E_6_+PADRE-J8_size distribution by number

L_15_E_6_+PADRE-J8_size distribution by intensity

L_15_E_6_+PADRE-J8_zeta potential


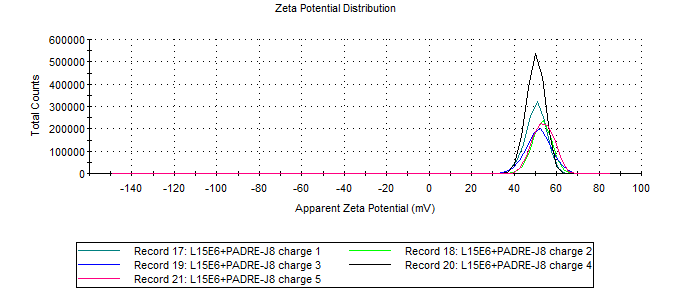


OVA_size distribution by intensity

OVA_size distribution by number


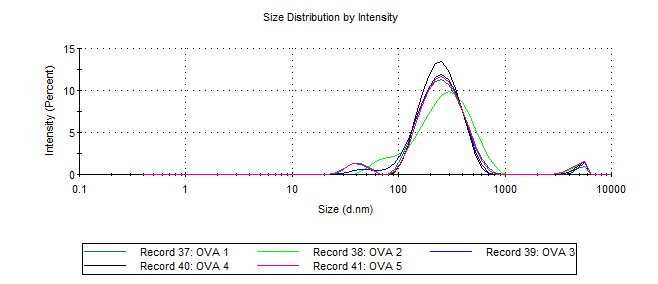

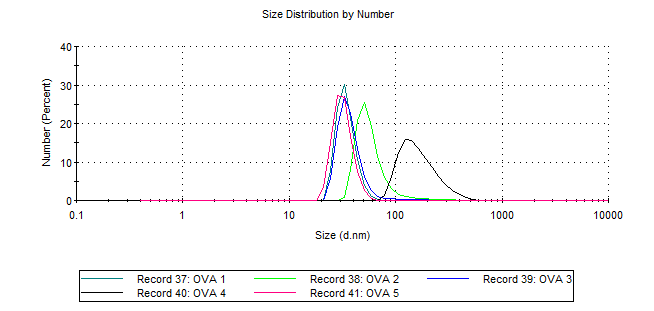


OVA_zeta potential


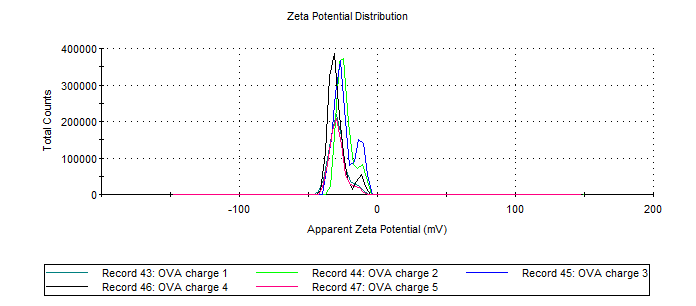


L_15_K_6_+OVA(5:20)_size distribution by intensity

L_15_K_6_+OVA(5:20)_size distribution by intensity


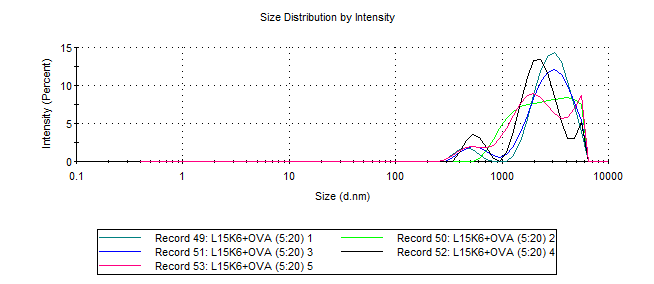

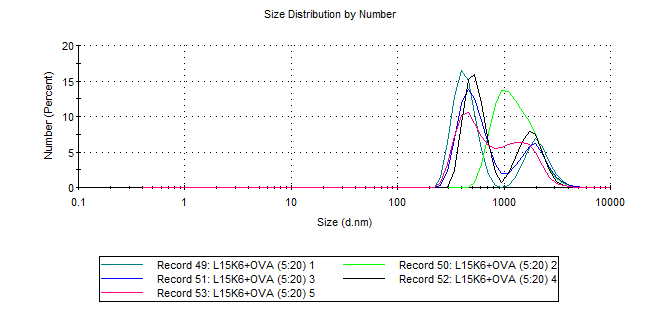


L_15_K_6_+OVA(5:20)_zeta potential


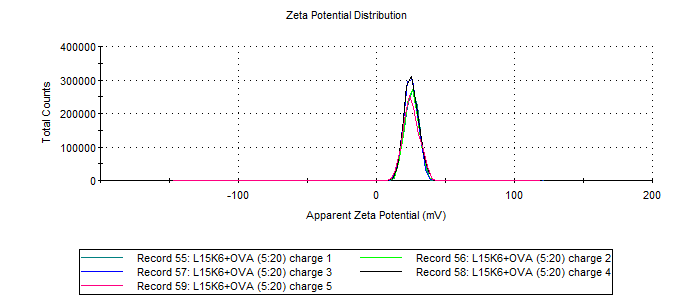


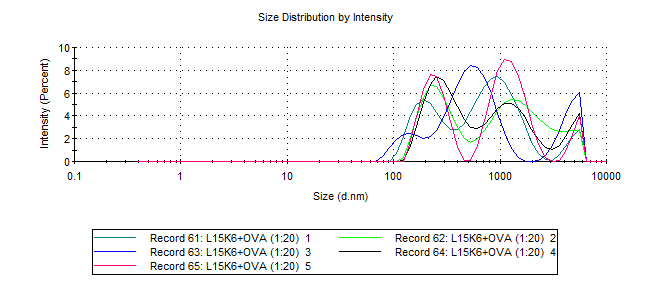

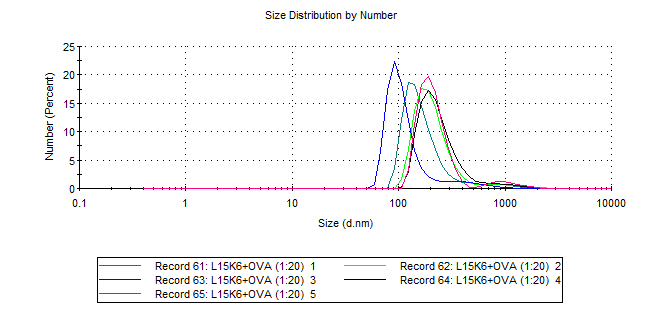


L_15_K_6_+OVA(1:20)_zeta potential

L_15_K_6_+OVA(1:20)_size distribution by intensity

L_15_K_6_+OVA(1:20)_size distribution by intensity


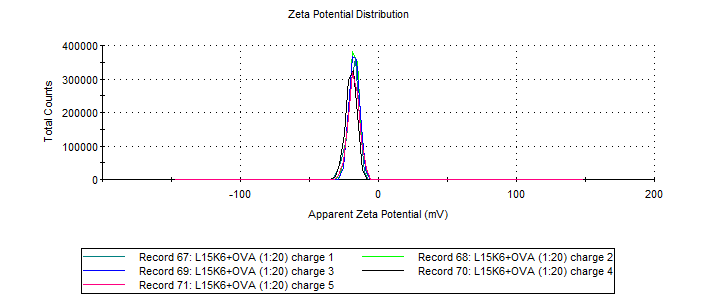


BSA_size distribution by number

BSA_size distribution by intensity


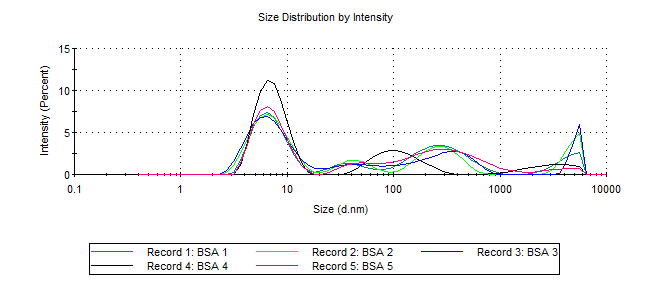

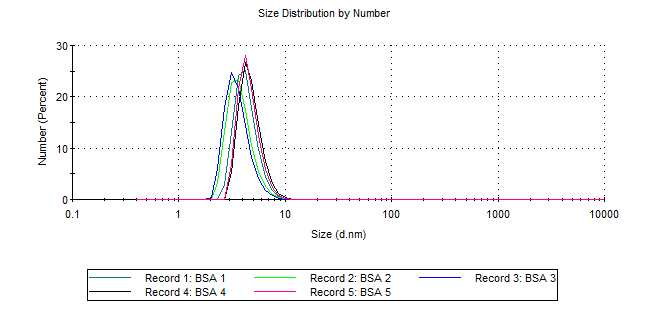


BSA_zeta potential


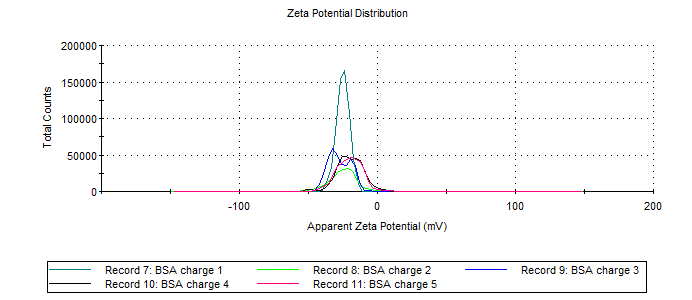


L_15_K_6_+BSA_size distribution by number

L_15_K_6_+BSA_size distribution by intensity


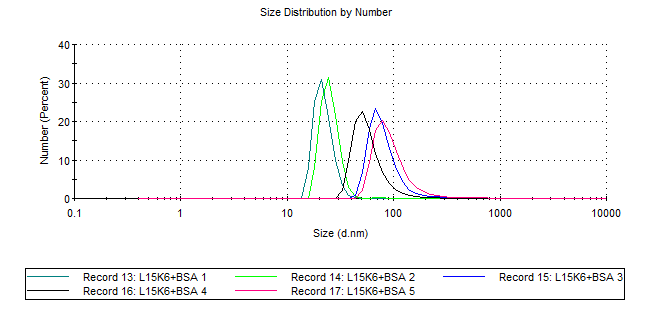

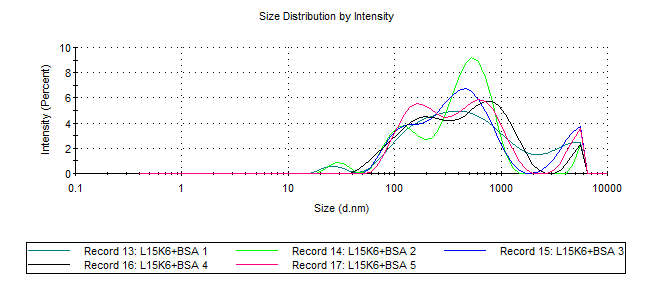


L_15_K_6_+BSA_zeta potential


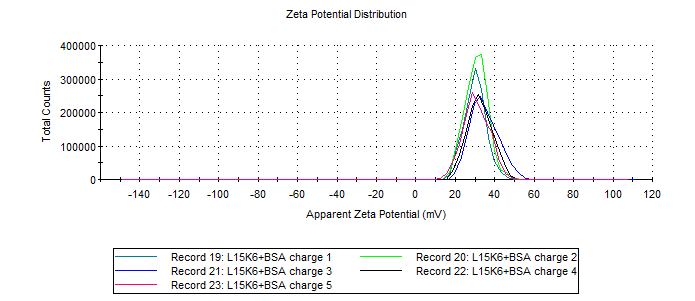


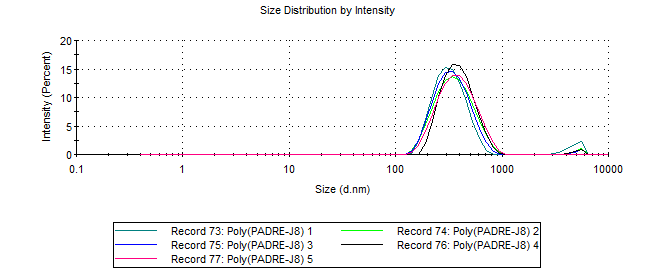

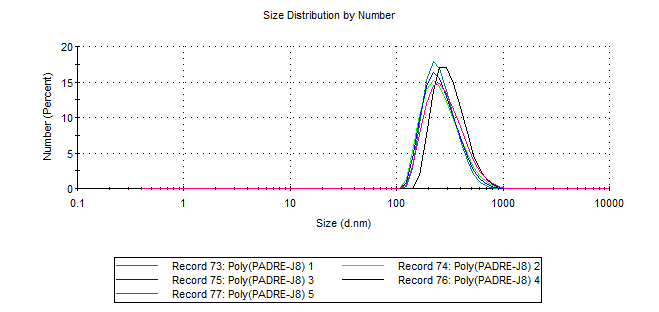


Poly(PADRE-J8)_size distribution by charge

Poly(PADRE-J8)_size distribution by number

Poly(PADRE-J8)_size distribution by intensity


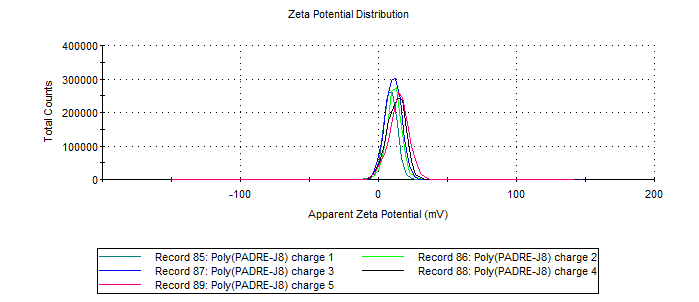


L_15_K_6_+poly(PADRE-J8)_size distribution by intensity

L_15_K_6_+poly(PADRE-J8)_size distribution by number


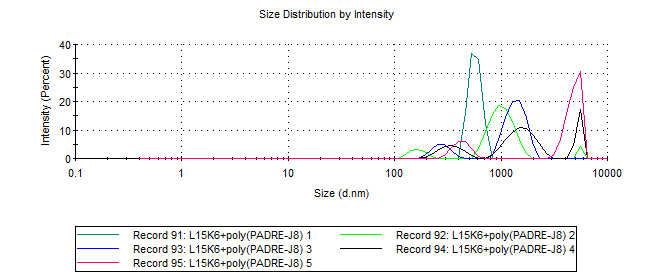

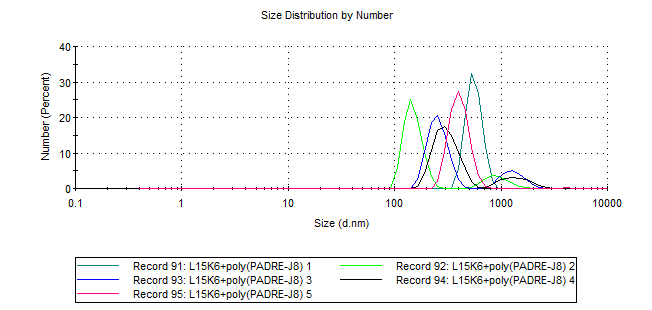


L_15_K_6_+poly(PADRE-J8)_zeta potential


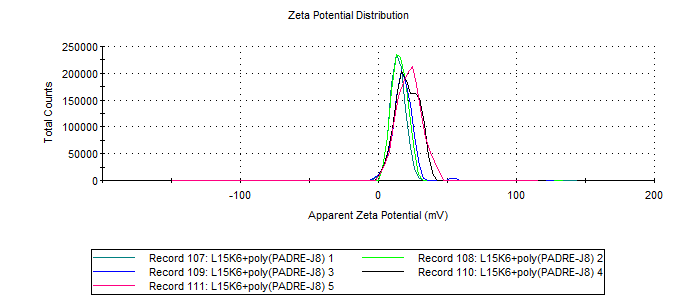


**Supplementary Figure 3**. DLS characterization of nanoparticles formed by polyleucine derivatives.

25 kDa

37 kDa

**Supplementary Figure 4.** Characterization of poly(PADRE-J8). The molecular weight of poly(PADRE-J8) was between 25 kDa and 37 kDa.


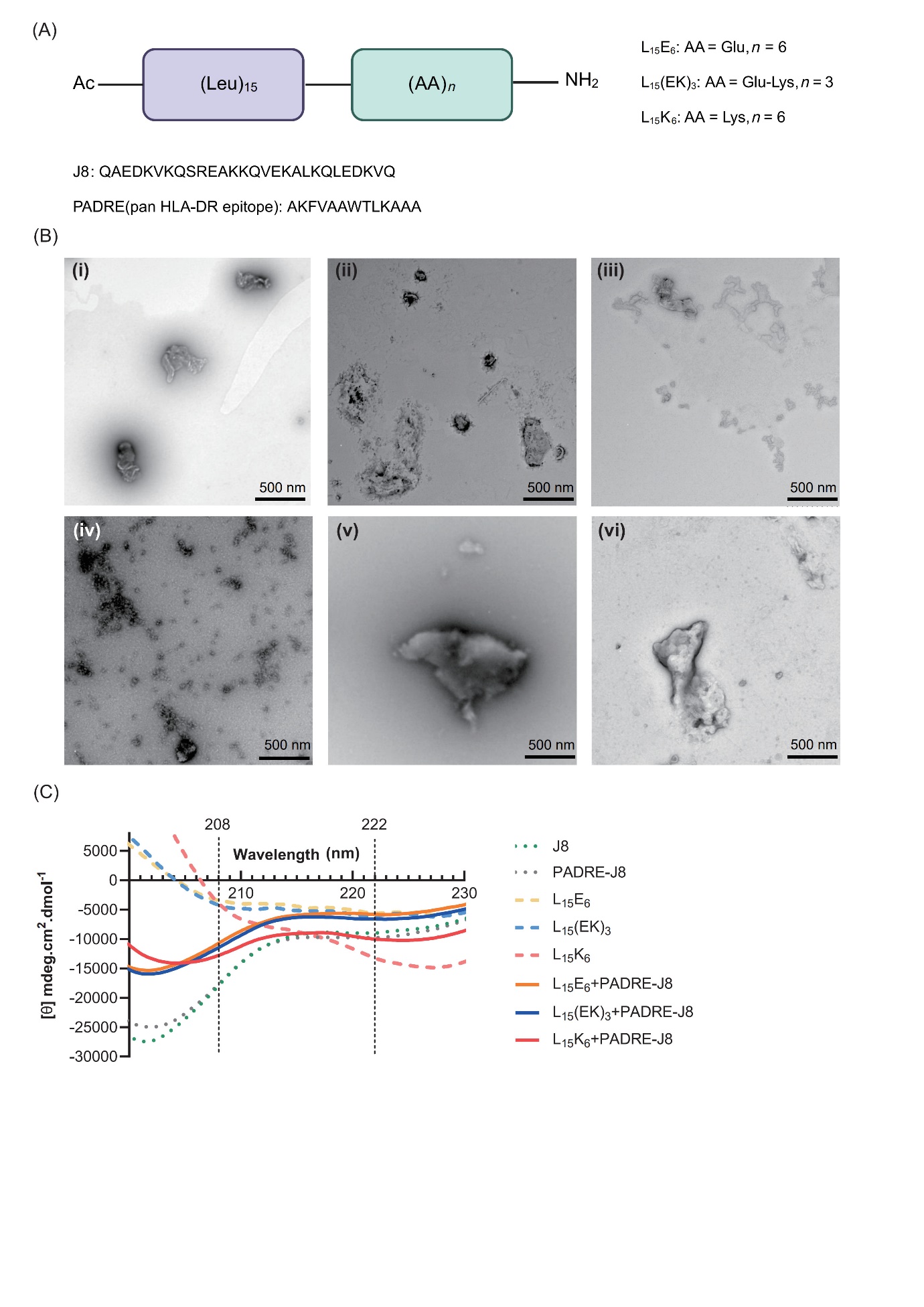


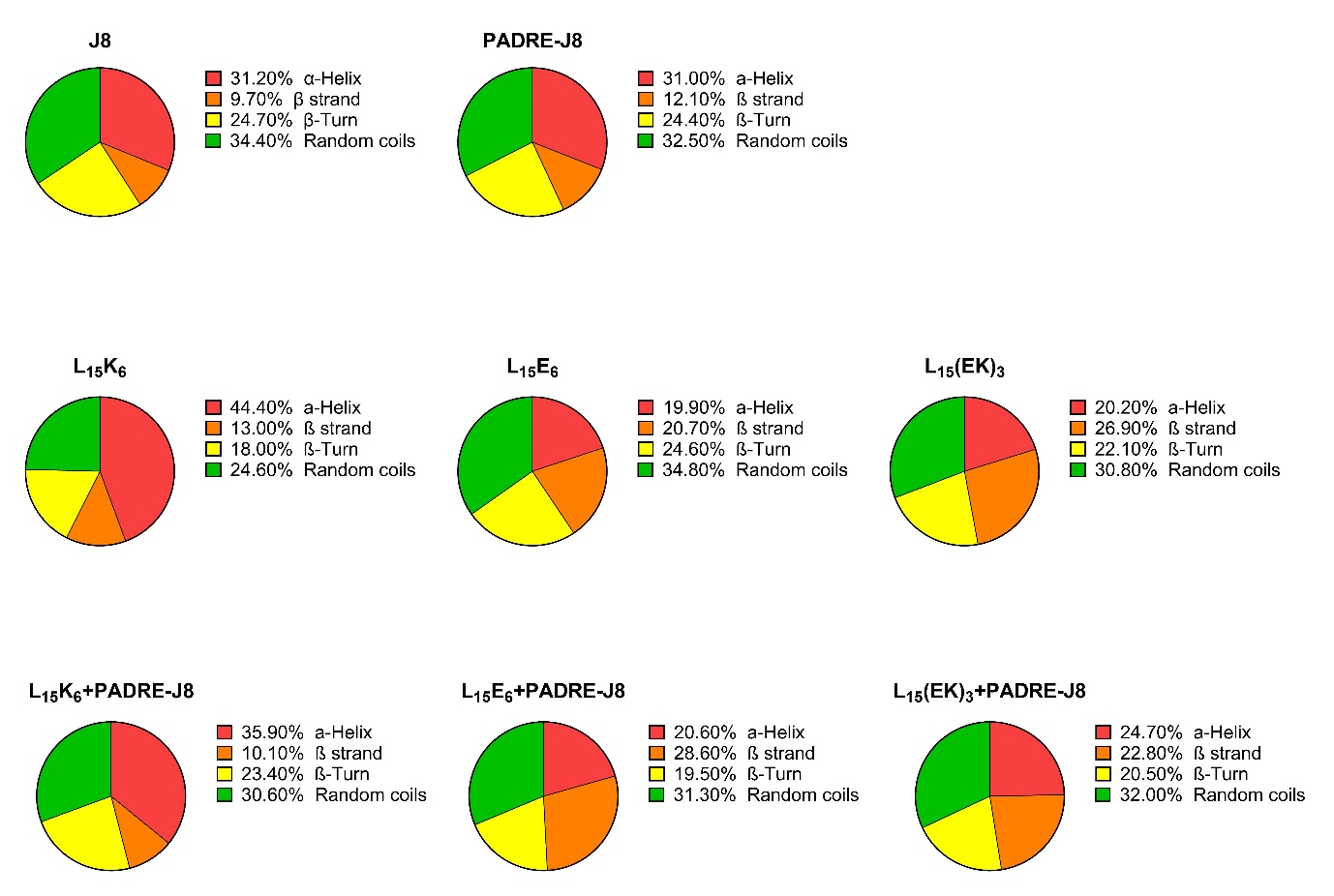


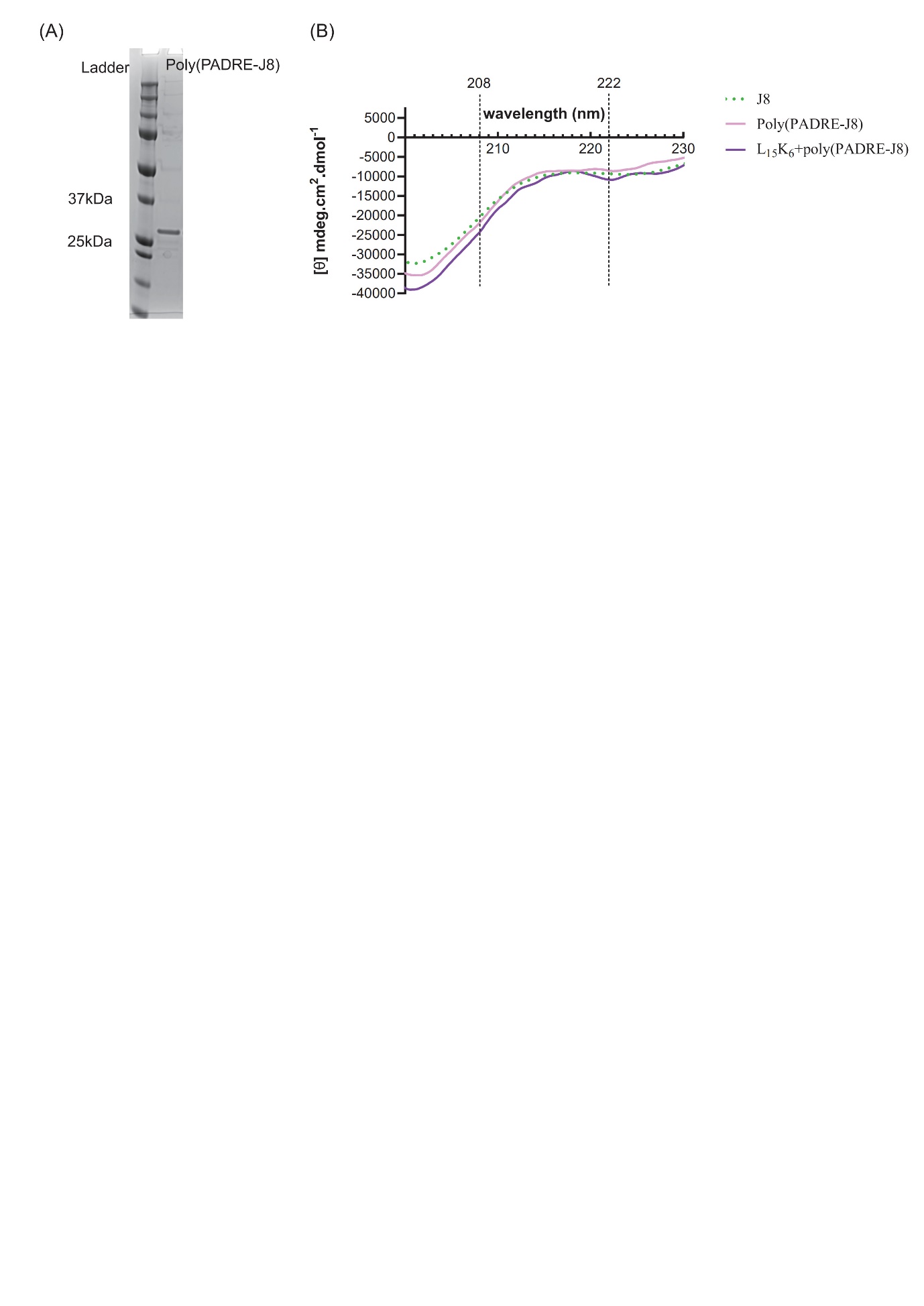


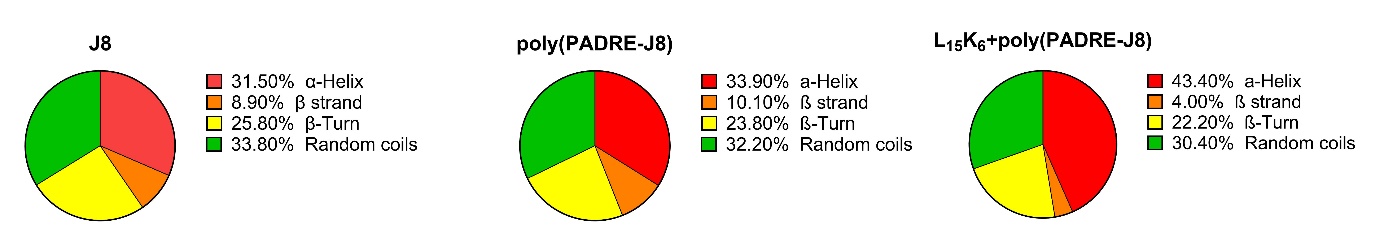


**Supplementary Figure 5.** CD spectroscopy of polyleucine derivatives and their physical mixtures with different antigens.


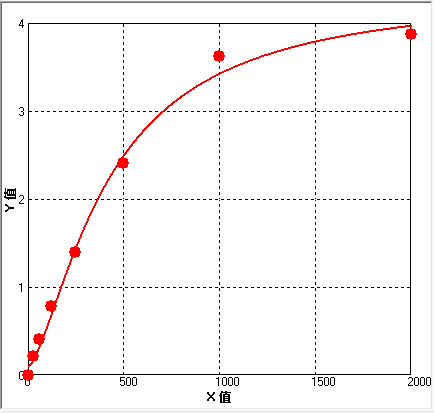

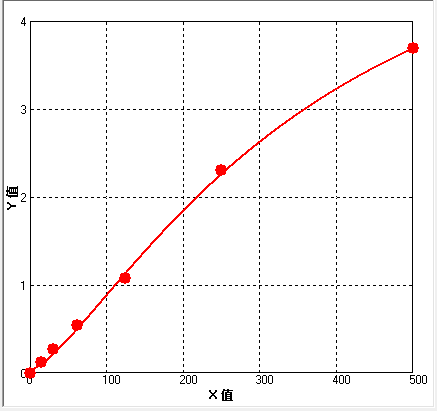


**X (concentration, pg/mL)**

**X (concentration, pg/mL)**

**Mouse IL-6**

**Four parameter logistic regression (4PL)**

Y = (A-D)/[1+(X/C)^B]+D

A = 6.12239

B = -1.37850

C = 371.52390

D = 0.03417

r^2^ = 0.99921

**Mouse TNF-α**

**Four parameter logistic regression (4PL)**

Y = (A-D)/[1+(X/C)^B]+D

A = 4.35450

B = -1.46980

C = 419.74826

D = 0.09548

r^2^ = 0.99520

**Y (Calibrited absorbance)**

**Y (Calibrited absorbance)**


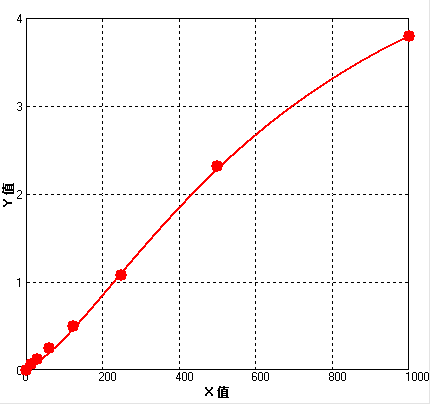

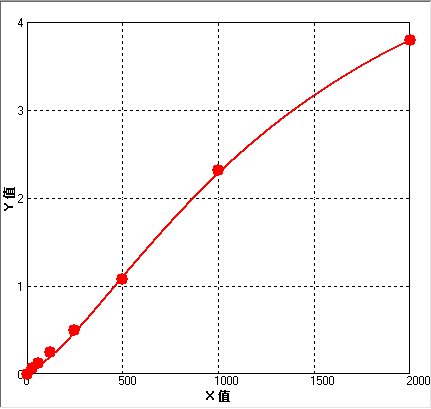


**X (concentration, pg/mL)**

**X (concentration, pg/mL)**

**Y (Calibrited absorbance)**

**Mouse IL-10**

**Four parameter logistic regression (4PL)**

Y = (A-D)/[1+(X/C)^B]+D

A = 6.17957

B = -1.44941

C = 1458.23294

D = 0.03768

r^2^ = 0.99960

**Mouse IL-4**

**Four parameter logistic regression (4PL)**

Y = (A-D)/[1+(X/C)^B]+D

A = 6.17955

B = -1.44942

C = 729.11347

D = 0.03768

r^2^ = 0.99960

**Y (Calibrited absorbance)**


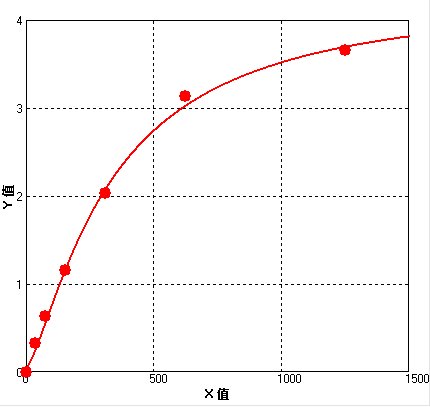

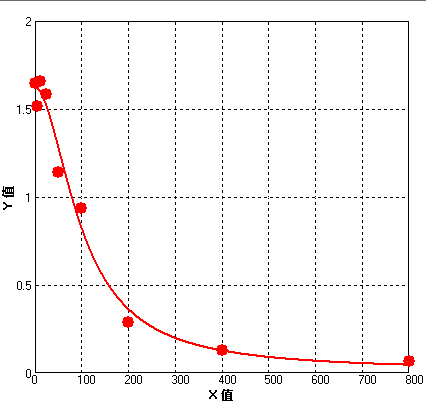


**Mouse C3a**

**Four parameter logistic regression (4PL)**

Y = (A-D)/[1+(X/C)^B]+D

A = 1.62246

B = 1.85695

C = 99.97068

D = 0.01145

r^2^ = 0.9836

**X (concentration, ng/mL)**

**Y (Calibrited absorbance)**

**Mouse IL-12 p70**

**Four parameter logistic regression (4PL)**

Y = (A-D)/[1+(X/C)^B]+D

A = 4.31328

B = -1.33879

C = 332.99829

D = 0.04754

r^2^ = 0.99807

**Y (Calibrited absorbance)**

**X (concentration, pg/mL)**

**Supplementary Figure 6.** ELISA standard curves.


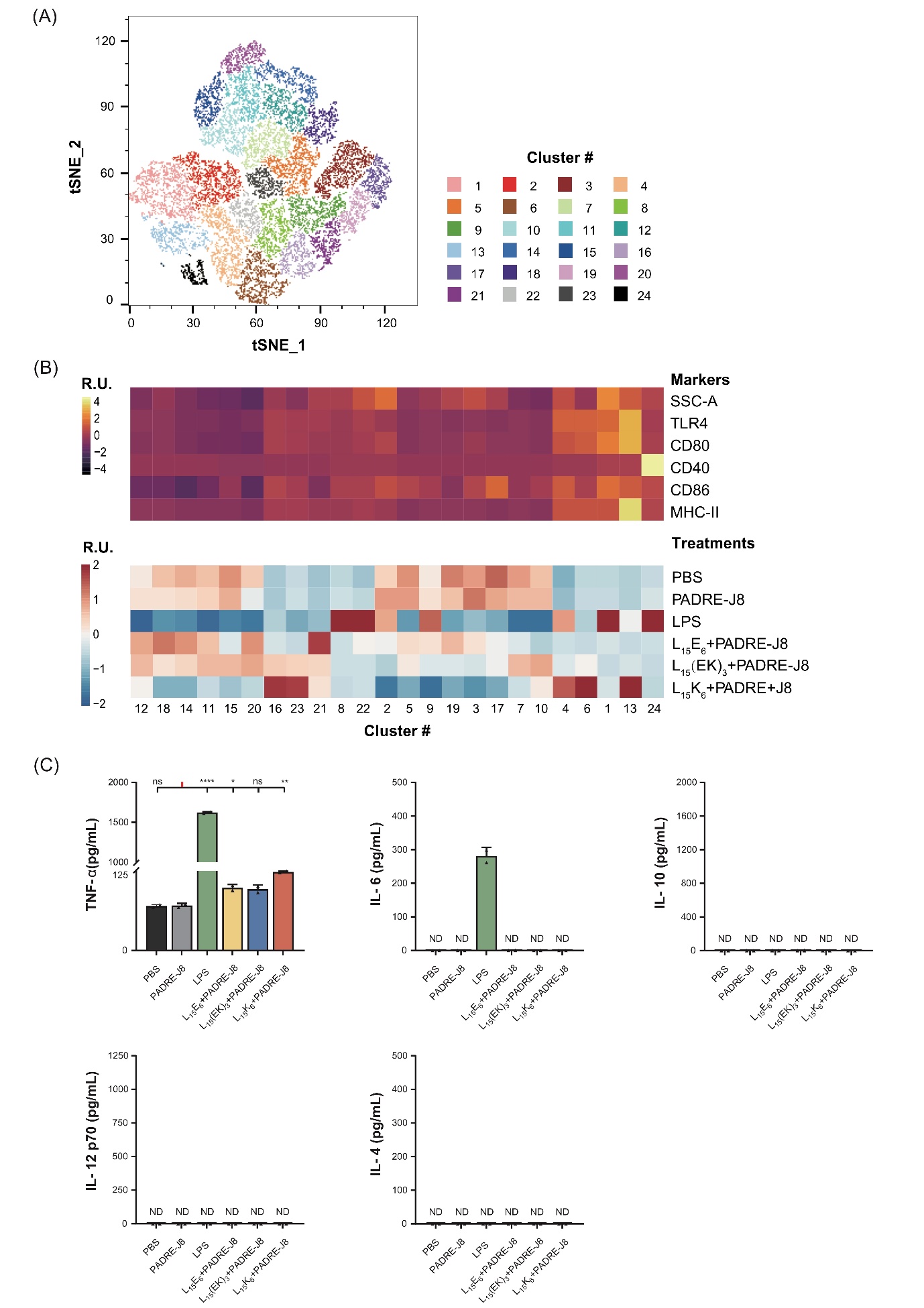

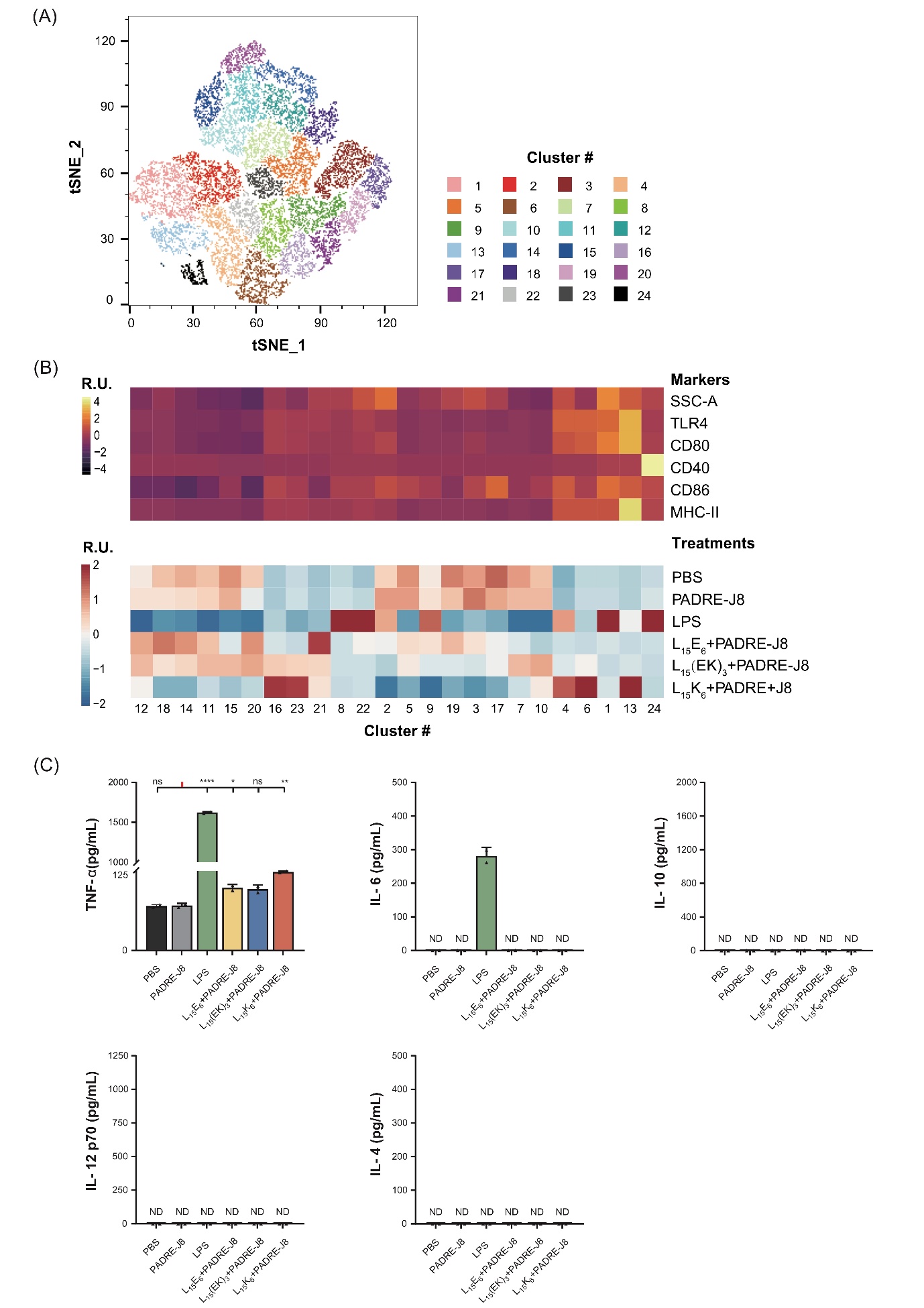


**Supplementary Figure 7.** Cytokine profiling for IL-12 p70, IL-4, and IL-10. ND: not detectable.


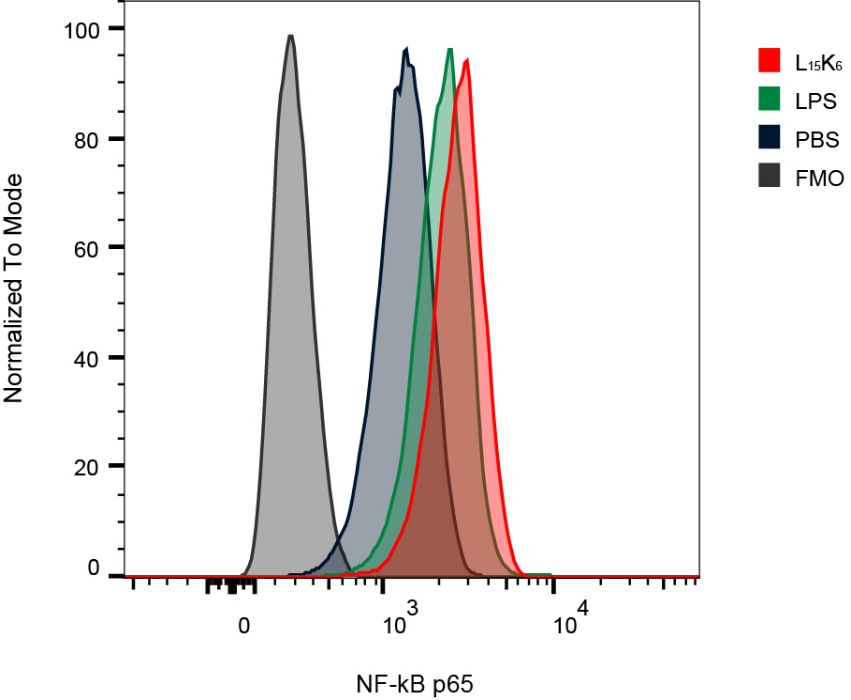


**Supplementary Figure 8.** L_15_K_6_ upregulated intracellular NF-κB p65 in DC2.4 cells.


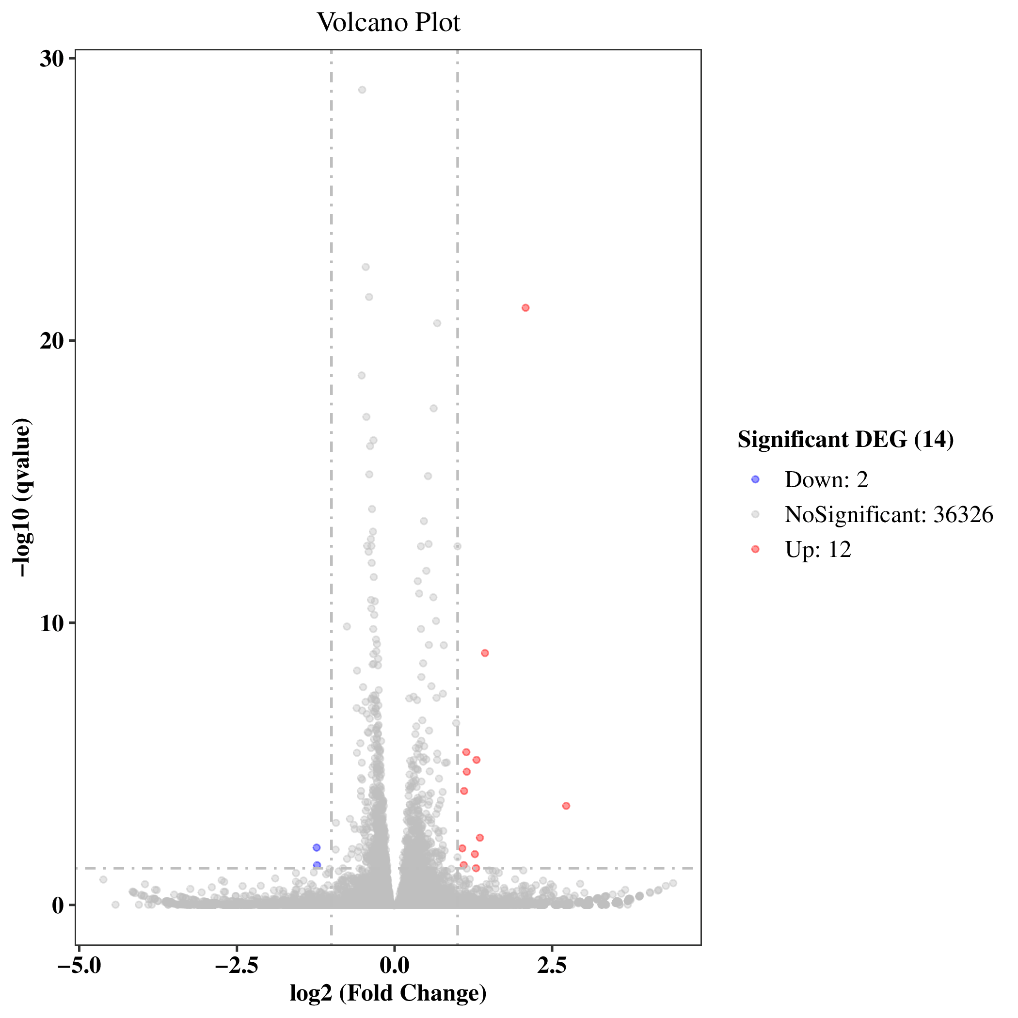


**Supplementary Figure 9.** Volcano plot of differentially expressed genes (DEGs) from bulk RNA sequencing analysis on THP-1 cells treated by PBS vs. L_15_K_6_. Up to 12 genes were significantly upregulated in THP-1 cells post the exposure to L_15_K_6_.


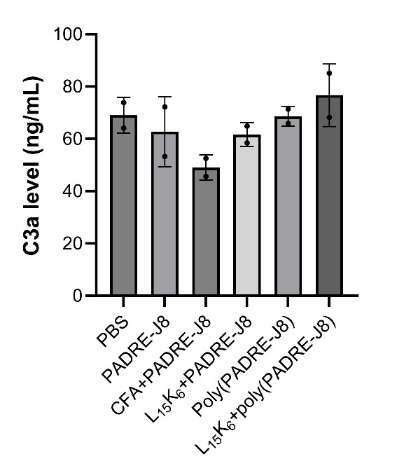


**Supplementary Figure 10.** C3a expression amongst mice receiving different treatments.
